# Phytochemical profiling and multi-target pharmacological evaluation of *Symphyotrichum squamatum* unveils its potential as a source of antidiabetic and anti-inflammatory agents

**DOI:** 10.3389/fchem.2026.1734411

**Published:** 2026-04-13

**Authors:** Mohammed Hassan, Islam Mostafa, Ahmed M. Saad, Ahmed S. AbdelKhalek, Mahmoud M. Elaasser, Zepeng Yin, Mahmoud Sitohy, Mohamed El-Sadek, Omer I. Fantoukh, Assem M. El-Shazly, Mona Fekry

**Affiliations:** 1 Department of Pharmacognosy, Faculty of Pharmacy, Zagazig University, Zagazig, Egypt; 2 Pharmaceutical Services Center, Faculty of Pharmacy, Zagazig University, Zagazig, Egypt; 3 Clinical Pharmacy Program, Medical Sector, Zagazig National University, 10^th^ of Ramadan City, Egypt; 4 Biochemistry Department, Faculty of Agriculture, Zagazig University, Zagazig, Egypt; 5 Department of Medicinal Chemistry, Faculty of Pharmacy, Zagazig University, Zagazig, Egypt; 6 Regional Center for Mycology and Biotechnology, Al-Azhar University, Cairo, Egypt; 7 Key Laboratory of Fruit Postharvest Biology of Liaoning Province, Shenyang, China; 8 College of Horticulture, Shenyang Agricultural University, Shenyang, China; 9 Department of Pharmacognosy, College of Pharmacy, King Saud University, Riyadh, Saudi Arabia; 10 Faculty of Pharmacy, El Saleheya El Gadida University, El Saleheya El Gadida, Egypt

**Keywords:** antidiabetic, anti-inflammatory, antioxidant, asteraceae, cytotoxicity, LC-MS/MS, *Symphyotrichum squamatum*

## Abstract

**Introduction:**

This study aimed to thoroughly profile the phytochemicals and pharmacological properties of *Symphyotrichum squamatum's* aerial parts to assess its potential as a valuable source of natural therapeutic agents. The research focused on isolating and identifying bioactive compounds and evaluating their antioxidant, cytotoxic, antidiabetic, and anti-inflammatory effects.

**Method:**

Chromatographic purification, LC/MS, total flavonoids, total phenolics, molecular docking and different pharmacological activity were used in this study to identify qualitatively and qualitatively the active compounds in extract and its activities.

**Results:**

Chromatographic purification of the petroleum ether and ethyl acetate fractions led to the isolation of four compounds: 3α- Friedelinol, Spinasterol, Dioctyl phthalate, and Kaempferol-3,7- diglucopyranoside. Additionally, the LC/MS profile of the hydro-alcoholic extract identified 35 metabolites, indicating a diverse chemical profile rich in fatty acids, phenolic propanoids, and terpenoids. Quantitative assays confirmed the extracts abundance in phenolics (65.9 ± 3.6 mg GAE/g extract) and flavonoids (25.8 ± 1.1 mg QE/g extract), correlating with notable in vitro antioxidant activity, as shown by low SC_50_ values of 77.00 μg/mL (DPPH) and 66.00 μg/mL (ABTS). The extract exhibited weak cytotoxicity against Hep-G2 and Panc-1 cell lines. Notably, both the extract and the isolated Kaempferol-3,7- diglucopyranoside demonstrated potent, dose-dependent inhibition of key carbohydrate-digesting enzymes, indicating antidiabetic activity. The flavonoid glycoside was particularly effective against α-amylase (IC_50_ = 24.29 μg/mL). The extract also showed promising anti-inflammatory activity via COX-1 inhibition (IC_50_ = 137.51 μg/mL). To explain these bioactivities, molecular docking studies were performed, revealing that the essential compounds, namely Kaempferol-3,7-diglucopyranoside and dicaffeoylquinic acids, form stable, high-affinity interactions with the reactive sites of α-amylase, α-glucosidase, and COX enzymes.

**Discussion:**

These findings collectively support *S. squamatum* as a promising candidate for further development in the management of diabetes and inflammation.

## 1 Introduction

*Symphyotrichum squamatum* is one of the Aster species plants belonging to family Asteraceae. These plants are known for production of a variety of secondary metabolites such as monoterpenes, diterpenes, sesquiterpenoids, flavones, coumarins, polyacetylenes, triterpene glycosides, phenolics, saponins, peptides, benzofurans, and esters (Awuchi and Morya, 2023). Traditionally, many Aster species have been used in folk medicine to treat respiratory conditions like bronchitis, pertussis, and pneumonia, as well as inflammatory disorders, skin infections, and fever (Pantoja and Oliveira, 2024). Scientific research supports these traditional uses, showing that Aster extracts have antifungal, antitumor, antioxidant, anti-inflammatory, and cytotoxic properties (Fan et al., 2024).

From the phytochemical point of view, the chemical composition of *S. squamatum* remains underexplored, with only limited research addressing its essential oil (EO) profile (Abd-ElGawad et al., 2020). Two published studies, one on Turkish *S. squamatum* and another on Japanese specimens, revealed distinct chemotypic differences (Abd-ElGawad et al., 2020). The Turkish ecotype was rich in sesquiterpene elemol and hexadecanoic acid, while the Japanese ecotype contained β-pinene and p-cymene as major components. Factors such as plant origin, developmental stage, harvest season, altitude, soil composition, and genetic background influence these chemical variations.

Phytochemical screening of *S. squamatum* has shown the presence of steroids, triterpenes, flavonoids, terpenes, phenols, amino compounds, saponins, and tannins. These molecules are associated with the plant’s reported antiulcer, antidiarrheal, antibacterial, antioxidant, and cytotoxic properties (Jeon et al., 2012; Gümrükçüoğlu, 2025; Hendawy et al., 2025).

The initial data highlighting the phytochemical potential of *S. squamatum*, its antioxidant, allelopathic, and biological properties, remain poorly characterized. Moreover, studies linking the identified bioactive compounds to their specific biological targets via molecular modeling remain limited. There is a significant research gap in the detailed characterization of the active compounds of *S. squamatum* and their associated biological mechanisms. Most current studies focus on general phytochemical screening or essential oil profiling, with limited attention to isolating, structurally elucidating, and bioactivity-guided fractionating specific metabolites. As a result, the pharmacological basis for its traditional therapeutic uses is still largely unclear. A systematic approach that combines advanced chromatographic, spectroscopic, and computational techniques is needed to identify the main active components and understand their biological mechanisms (Hassan et al., 2023; Salman et al., 2022).

Molecular docking analysis has become a crucial *in silico* method for studying how plant-derived compounds interact with target enzymes or receptors involved in oxidative stress, inflammation, and cancer development (Alshehri, 2025). In this investigation, molecular docking techniques were utilized to forecast the binding strengths and interaction profiles of phytochemical constituents identified from *S. squamatum* with critical target proteins, specifically α-amylase, α-glucosidase, cyclooxygenase-1 (COX-1) and cyclooxygenase-2 (COX-2). This computational approach helps uncover potential molecular mechanisms underlying the observed antioxidant, antidiabetic, and anti-inflammatory effects. By combining phytochemical profiling with docking-based target validation, this research offers a thorough understanding of the bioactivity potential and structure–function relationships of *S. squamatum* metabolites.

Therefore, the current study aimed to analyze the chemical composition of the aerial parts of *S. squamatum* and evaluate their antioxidant, cytotoxic, antidiabetic, and anti-inflammatory activities, supported by molecular docking studies to identify potential bioactive lead compounds.

## 2 Materials and methods

### 2.1 Plant collection, extraction, fractionation, and isolation

The aerial parts of *Symphyotrichum squamatum* were collected from the edges of a field beside the Cairo-Alexandria agricultural road in Qaluibya province, (30°27′17″N 31°11′51″E), Egypt. The plant identification was confirmed by Prof. Dr. Abd El-Halim Abd El-Mogali Mohamed from the Flora and Phytotaxonomy Research Department at HRI, ARC. A voucher specimen (S. sq. A) was deposited in the herbarium of the Pharmacognosy Department, Faculty of Pharmacy, Zagazig University, Egypt.

The plant material was air-dried and ground into powder, yielding 5 kg. The powder was extracted by cold maceration with 80% aqueous ethanol until complete exhaustion (5 extractions, 3 days and 12 L each). The combined extracts were concentrated under reduced pressure at 50 °C to yield 445 g of a dark green, viscous extract. About 400 g of the aerial parts’ extract was dissolved in a methanol-water mixture (1:9) and then fractionated using light petroleum (60-80 °C) (7 × 500 mL), dichloromethane (6 × 500 mL), and ethyl acetate (5 × 500 mL). The different fractions were concentrated under reduced pressure at 50 °C, yielding 28 g, 7 g, and 15 g of light petroleum, dichloromethane, and ethyl acetate fractions, respectively.

Light petroleum- and ethyl acetate-soluble fractions were separated using silica-gel column chromatography with gradient elution. The gradient began with 100% petroleum ether and shifted to 100% methylene chloride, then increased to 100% methanol for the petroleum ether fraction. For the ethyl acetate fraction, the gradient started with 100% petroleum ether and moved to 100% ethyl acetate, then reached 100% methanol. Additionally, the total hydro-alcoholic extract was analyzed using HPLC-PDA-ESI-MS/MS.

### 2.2 LC/MS profile of active compounds

To ensure the chemical integrity of the isolates and mitigate potential laboratory artifacts, a rigorous contamination control protocol was implemented. All extraction and purification steps were performed using borosilicate glassware pre-rinsed with HPLC-grade *n*-hexane and acetone. The use of polypropylene (PP) or polyethylene (PE) materials was strictly avoided during critical sample concentration steps. A procedural blank was processed in parallel with the *Aster* plant material.

A solution of the aerial parts’ hydroalcoholic extract was prepared at a concentration of 100 μg/mL using high-performance liquid chromatography (HPLC)-grade methanol. It was then filtered through a 0.25 μm membrane disc filter and stored at 4 °C until analysis. HPLC-ESI-MS/MS analysis was performed in both positive and negative ion modes using a XEVO TQD mass spectrometer from Waters Corporation, Milford, MA, United States. Ten microliters of the prepared solution were injected into an HPLC system equipped with a reversed-phase C-18 column (ACQUITY UPLC-BEH C18, 1.7 μm, 2.1 × 50 mm). The mobile phase was delivered at a flow rate of 0.2 mL/min via gradient elution, consisting of Eluent A (90% water with 10% acetonitrile) and Eluent B (methanol acidified with 0.1% formic acid). Both eluents were filtered through 0.25 μm membrane disc filters and degassed by sonication before use. The gradient elution program was as follows: 90% A/10% B (0–2 min), 70% A/30% B (5 min), 30% A/70% B (15 min), 10% A/90% B (25 min), returning to 90% A/10% B (27 min). The source temperature was set to 150 °C, cone voltage to 30 eV, capillary voltage to 3 kV, desolvation temperature to 440 °C, cone gas flow to 50 L/h, and desolvation gas flow to 900 L/h. Mass spectra were acquired across the range of 100–1,000 m/z. The LC-MS data were processed using MassLynx 4.1, where ApexTrack detection was selected for its superior noise discrimination, coupled with minimal (1–2-point) smoothing to preserve authentic peak shape. Adaptive background subtraction corrected for baseline drift, and a collision energy ramp (10–40 eV) during MS/MS ensured comprehensive fragmentation across compounds of varying stability. Identifications were established by matching experimental data to the FooDB library and literature using a multi-tiered approach: a flexible m/z tolerance (±0.1–0.5 Da/5–10 ppm) accommodated instrument performance across the mass range, a strict retention time window (±0.2 min) ensured chromatographic reliability, and spectral matches were required to exceed thresholds for score (>80%), fit (i-Fit >90%), and signal quality (S/N > 10). This rigorous identification was validated through technical triplicates (RSD <5%) to ensure precision and monitored using a reserpine quality control standard (S/N > 30,000:1) to confirm sustained instrument sensitivity and stability, with IntelliStart automated calibration applied pre-run to guarantee reproducibility. To explore bioactivity, molecular docking simulations—exemplified by kaempferol-3,7-diglucopyranoside against the COX-1 enzyme (PDB: 3N8Y)—were performed with AutoDock Vina. The protocol was validated by successfully re-docking the native ligand (RMSD <2.0 Å), confirming the reliability of the chosen parameters: a grid box centered on catalytic residues (Tyr385, Ser530) to focus computational effort on the active site, an exhaustiveness of 8 to balance accuracy and speed, and the generation of 9 binding modes per compound for analysis. The top-ranked docked poses then underwent 100-ns molecular dynamics simulations using GROMACS to assess binding stability in a dynamic environment, with stable complexes defined by an RMSD plateau below 3 Å, residue fluctuations (RMSF) under 2.5 Å in the binding site, and a calculated binding free energy (MM/GBSA ΔG) more favorable than −20 kcal/mol. Finally, to evaluate pharmacokinetic potential, SwissADME was employed for ADMET prediction, applying Lipinski’s rule-of-five (MW < 500 Da, logP <5, HBD ≤5, HBA ≤10) as an initial filter for drug-likeness, ensuring that the computationally identified candidates possessed physiologically relevant properties.

### 2.3 Total phenolics and total flavonoids determination

The total phenolic content of *S. squamatum* aerial part extract (SSAE) was determined using the Folin–Ciocalteu method. Briefly, 1 mL of SSAE was mixed with 2 mL of Folin–Ciocalteu reagent and incubated at room temperature for 15 min. Subsequently, 2 mL of Na_2_CO_3_ solution was added to the mixture to develop a blue coloration. After 30 min incubation, the color was read at 765 nm using a spectrophotometer (Saad et al., 2021). Gallic acid (GA) was used as a standard, and results were calculated as mg GAE/g extract.​

For total flavonoids, the AlCl_3_ colorimetric assay was utilized (Nam et al., 2022). Extracted samples were combined with an aluminum chloride solution and, after suitable incubation, the resulting yellow hue was measured spectrophotometrically (typically at 415 nm). A quercetin calibration curve was generated, and flavonoid content was expressed as mg QE/g extract.

### 2.4 Antioxidant activity

#### 2.4.1 DPPH assay

The scavenging activity of SSAE aganist DPPH (2,2-diphenyl-1-picrylhydrazyl) radical were adapted from Bhakya et al. with slight modifications. SSAE concentrations at 50, 100, 200, and 400 μg/mL were prepared in ethanol. 100 μL of SSAE was combined with 100 μL of a freshly prepared DPPH solution (0.1 mM in ethanol) in individual wells of a microtiter plate. The mixture was incubated for 30 min to allow maximum interaction between antioxidants in the extract and the DPPH radicals.

Following incubation, the absorbance of each well was measured at 517 nm using a microtiter plate reader (BioTek Elx808, United States), with ethanol as the blank and ascorbic acid as the positive control. DPPH solution without extract was used as a negative control. The percentage of radical scavenging activity was computed using the equation:

$$\%\;Antioxidant\;activity=\left[\frac{\left(Absorbance\;control-Absorbance\;sample\right)}{Absorbance\;control}\right]\times 100$$

#### 2.4.2 ABTS assay

The method outlined by Gil et al. (2002) was used to determine the antioxidant activity of the aerial parts’ extract using the ABTS assay, with a few modifications. To evaluate the ABTS radical scavenging activity, 100 μL of SSAE concentrations (50, 100, 200, and 400 μg/mL) was added to 100 μL of freshly prepared 0.1 mM ABTS solution in each well of a microtiter plate. The reaction mixtures were incubated to ensure complete interaction between antioxidants in the extract and ABTS radicals. After incubation, absorbance was recorded at 745 nm using a microtiter plate reader (BioTek Elx808, United States). Ascorbic acid (AsA) served as a positive control, and the ABTS solution without extract served as a negative control. The % inhibition of free radicals was calculated using the previous equation.

### 2.5 Cytotoxic activity

The cytotoxic effect of the *Symphyotrichum squamatum* aerial parts extract (SSA extract) on liver and pancreatic cancer cell lines was evaluated using the MTT assay, a widely accepted colorimetric method for assessing cell viability based on mitochondrial activity. The cell lines present in this study included HepG2 and PANC-1, representative liver and pancreatic cancer cell lines, respectively. These cell lines were obtained from the American Type Culture Collection (ATCC, Manassas, VA, United States). Both HepG2 and PANC-1 were cultured and seeded into 96-well plates at an appropriate density (10,000 cells per well) and allowed to adhere for 24 h under standard incubator conditions (37 °C, 5% CO_2_). The cells were then exposed to different concentrations of the SSA extract for a specified period, usually 24 or 48 h.

Following treatment, 10 µL of MTT reagent (5 mg/mL) was added to each well, and the plates were incubated for 3–4 h to allow viable cells to convert the yellow tetrazolium salt into insoluble purple formazan crystals via mitochondrial dehydrogenase activity. After incubation, the culture medium was carefully removed, and 100–200 µL of dimethyl sulfoxide (DMSO) or a suitable solubilizing agent was added to dissolve the formazan crystals.

The absorbance of each well was measured using a microplate reader at 550–570 nm, and the optical density was correlated with the number of viable cells. Untreated cells served as controls to establish baseline viability. The reduction in absorbance in SSA extract–treated wells relative to controls was used to calculate cell viability and determine the cytotoxicity index of the extract (Hans et al., 1989).

#### 2.5.1 Caspase Colorimetric Assays

For caspases, particularly caspase-3/7, colorimetric kits use the synthetic peptide substrate DEVD-pNA, which is cleaved by active caspases in cell lysates or tissue extracts. The hydrolysis releases the yellow chromophore p-nitroaniline (pNA), which is quantified by absorbance at 405 nm using a spectrophotometer or microplate reader, allowing direct correlation of optical density to enzyme activity after normalization to protein content (via Bradford assay).

#### 2.5.2 Bcl-2 Colorimetric Assays

Bcl-2 quantification via colorimetric methods primarily uses cell-based ELISA kits, in which cells are fixed, permeabilized, and probed with anti-Bcl-2 antibodies, followed by a horseradish peroxidase (HRP)-conjugated secondary antibody. The addition of a chromogenic substrate, such as TMB, produces a colored product measured at 450 nm, enabling relative quantification of Bcl-2 protein in adherent or suspension cells.

### 2.6 Antidiabetic activity

#### 2.6.1 α-amylase inhibition assay

Stock solutions of the aerial part extract (A), kaempferol-3, 7-diglucopyranoside (K), and acarbose were prepared in water. Inhibition of porcine α-amylase activity was determined using dinitrosalicylic acid as described (KWON et al., 2008; Poovitha and Parani, 2016). In brief, 100 μL from each sample was added to 100 μL of α-amylase (1 U/ml) and 200 μL of sodium phosphate buffer (20 mM, pH 6.9) to get 2–1000 μg/mL final concentration. After that, 200 μL of 1% starch was added, followed by incubation at 25 °C for 10 min 1 mL of dinitrosalicylic acid was added to the reaction mixture, incubated in a boiling water bath for 5 min, then cooled to room temperature and diluted to a 1:5 ratio with water. The absorbance was measured at 540 nm, and the percentage of inhibition was calculated using the following equation:

$$\%\text{ Inhibition}=\left[\frac{\left(Absorbance\;control-Absorbance\;teatment\right)}{Absorbance\;control}\right]\times 100$$

The IC_50_ was calculated from the concentration response curve using GraphPad PRISM.

#### 2.6.2 α-glucosidase inhibition assay

Inhibition of α-glucosidase activity was measured for the aerial part extract (A), kaempferol-3,7-diglucopyranoside (K), and acarbose using yeast α-glucosidase and p-nitrophenyl-α-D-glucopyranoside (pNPG) was demonstrated (Kwon et al., 2008; Poovitha and Parani, 2016). Shortly, 100 μL from each sample was added to 50 μL of α-glucosidase (1 U/ml) prepared in 0.1 M phosphate buffer (pH 6.9), and 250 μL of 0.1 M phosphate buffer to get 2–1000 μg/mL final concentration. This was followed by the addition of 10 μL of 10 mM pNPG prepared in 0.1 M phosphate buffer (pH 6.9), then incubation at 37 °C for 30 min. The absorbance was measured at 405 nm and percentage of inhibition was calculated using the following equation:

$$\%\text{ Inhibition}=\left[\frac{\left(Absorbance\;control-Absorbance\;teatment\right)}{Absorbance\;control}\right]\times 100$$

### 2.7 Anti-inflammatory activity

The *in vitro* ability of the aerial part extract (A) and kaempferol-3,7-diglucopyranoside (K) to inhibit COX-1 isoenzymes was determined using a COX-1 inhibitor screening assay, as demonstrated by Alaa et al. (2016) and Elaasser et al. (2020). Different concentrations of the tested sample (3.9–2000 μg/mL were pre-incubated with the cyclooxygenase enzymes at 25 °C for 5 min in the presence of hematin. 500 μM phenol, 20 μM 1-leuco-dichlorofluorescein, 50 μM arachidonic acid, and 1 μM hematin in 1 mL of 0.1 M Tris-buffer were added to the enzyme mixture, then absorbance was measured at 502 nm using Celecoxib as a standard. The IC_50_ was calculated from the curve.

### 2.8 Molecular docking

The SMILES structures of the compounds identified from the aerial parts of *S. squamatum* were retrieved from the PubChem database (https://pubchem.ncbi.nlm.nih.gov). The 3D structure conformations were generated using the Molecular Operating Environment (MOE, version 2019.0102). Before molecular docking, the ligand structures were protonated and energy-minimized to achieve geometric stability. The crystal structures of α-glucosidase (PDB ID: 3W37), α-amylase (PDB ID: 3BAJ), cyclooxygenase-1 (COX-1; PDB ID: 1EQG) and cyclooxygenase-2 COX-2 (PDB ID: 1CX2). Were downloaded from the RCSB Protein Data Bank (https://www.rcsb.org). Protein preparation involved hydrogen atom addition, correction of missing residues, removal of crystallographic water molecules, and energy minimization, refined to a gradient RMS of 0.1 kcal/mol/Å^2^. Using identical parameters, the docking accuracy was confirmed. This involved the re-docking of the original co-crystallized ligands: acarbose for alpha-glucosidase and alpha-amylase, ibuprofen for COX-1, and SC-558 for COX-2. The re-docked ligands exhibited excellent alignment with their original crystallographic conformations, confirming the reliability of the docking protocol, with RMSD values below 2 Å (Supplementary Figure S1 in the supplementary data).

### 2.9 Statistical analysis

All experimental results for *S. squamatum* profiling and bioactivities were expressed as mean ± standard deviation (SD) of three independent replicates (*n* = 3). The normality of the data was assessed using the Shapiro-Wilk test. Statistical comparisons between different concentrations and the reference standards were performed using One-way Analysis of Variance (ANOVA). To identify specific differences between means, Tukey’s Honestly Significant Difference (HSD) post hoc test was applied.

For the pharmacological evaluation, the Half-maximal inhibitory concentration (IC_50_) was calculated via non-linear regression analysis. All statistical procedures were conducted using GraphPad Prism 10.6.1, and a p-value <0.05 was defined as the threshold for statistical significance. Significance levels are indicated in figures as *p* < 0.05.

## 3 Results

### 3.1 Characterization of the isolated compounds from petroleum ether and ethyl acetate fractions of *Symphyotrichum squamatum* aerial parts

**FIGURE 1:**
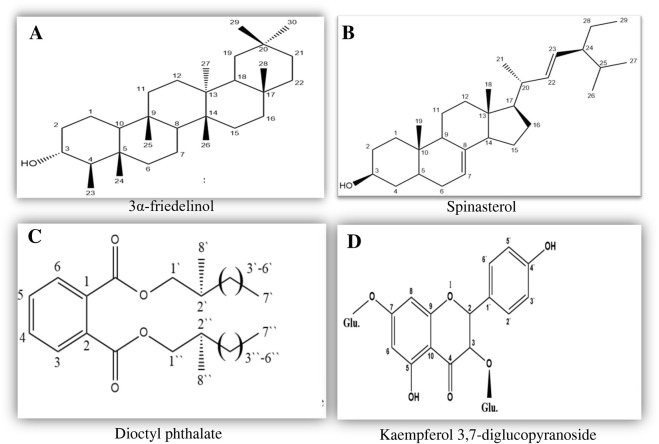
Chemical structures of the compounds isolated from the aerial parts of *Symphyotrichum squamatum*, including petroleum ether **(A,B)** and ethyl acetate fractions **(C,D)**.

Chromatographic analysis of petroleum ether and ethyl acetate fractions led to the isolation of compounds 1 and 2 from the petroleum ether fraction and compounds 3 and 4 from the ethyl acetate fraction. Structural elucidation was performed using UV, 1H-NMR, and 13C-NMR, in accordance with reported data. The chemical structures of the isolated compounds are presented in Figure 1. Compound 1 was obtained as a white crystalline material (140 mg) and exhibited an R_f_ value of 0.63 (light petroleum: ethyl acetate/6:4) and was identified as 3β-Friedelinol, supplemental data 1 [13]. Compound 2 was isolated as white crystals (237 mg) with an R_f_ value of 0.43 (light petroleum: ethyl acetate/6:4) and was identified as Spinasterol, supplemental data 1 [14]. Compound 3 was a colorless oil (70 mg) with an R_f_ value of 52 (light petroleum: ethyl acetate/4:6) and was identified as dioctyl phthalate, supplemental data 1 [15]. Compound 4 was a yellow amorphous powder (56 mg) with an R_f_ value of 0.6 (methylene chloride: methanol/9:1) and was identified as kaempferol 3,7-diglucopyranoside, supplemental data 1 [16–20].

### 3.2 Chemical constituents of the hydro-alcoholic extract of *Symphyotrichum squamatum* aerial parts

**FIGURE 2:**
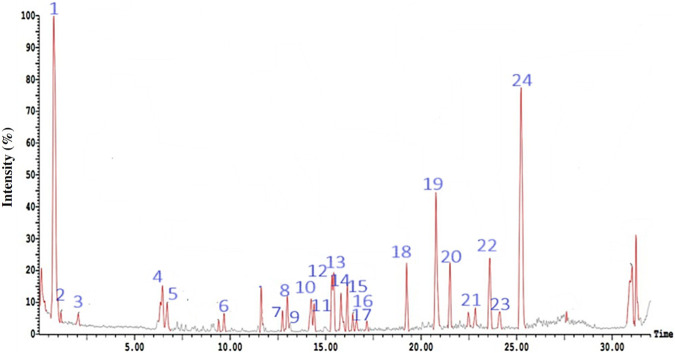
HPLC-ESI-MS chromatogram in positive ion mode of the hydro-alcoholic extract of the aerial parts of *Symphyotrichum squamatum*. Compounds corresponding to peak numbers are shown in Table 1.

**FIGURE 3 F3:**
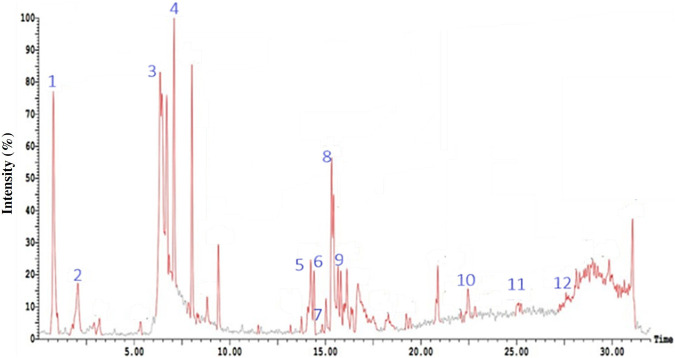
HPLC-ESI-MS chromatogram in negative ion mode of the hydroalcoholic extract of aerial parts of *Symphyotrichum squamatum*. Compounds corresponding to peak numbers are shown in Table 2.

HPLC-PDA-ESI-MS/MS analysis of the hydro-alcoholic extract in both positive and negative ion modes identified 24 and 11 metabolites, respectively (Figures 2, 3 [Fig F3]; Tables 1, 2). The compounds spanned a wide variety of secondary metabolite classes, including 8 fatty acids, 5 phenolic propanoids, 4 phenyl propanoids, 3 monoterpenes, 2 sesquiterpenes, 2 amino acids, 2 long-chain fatty alcohols, and one each of phenolics, flavonoids, coumarins, tetraterpenes, triterpenes, diterpenes, sterols, acetylenic fatty alcohols, and phenyl acetic acid. High-resolution Mass Spectrometry (HRMS) was used to characterize Compound 3. The mass spectrum of Compound 3 revealed a molecular ion peak at m/z 275 [M-H]^-^ (negative ion mode) and characteristic fragment ions at m/z 221, 167, and 95, consistent with the structure of Stearidonic acid (C18:4, omega-3). Crucially, common plastic-derived slip agents, such as erucamide (m/z 337) and stearamide (m/z 283), were absent in the isolated fraction and the procedural blank, confirming that Compound 3 is an endogenous metabolite of the *Aster* extract. The amino acid betaine and the sterol compound spinasterol were the dominant compounds in the positive ion chromatogram with areas of 24.84% and 18.99%, respectively, while the phenolic compounds 4,5-dicaffeoyl quinic acid and 5-caffeoyl quinic acid were the major components in the negative ion chromatogram with areas of 13.66% and 6.71%, respectively.

**TABLE 1:** Secondary metabolites tentatively identified from the aerial parts extract of *Symphyotrichum squamatum* in positive ionization mode.

No	M + H	Rt	MS^2^ (m/z)	Compound name	Class	References
1	118	0.78	118, 72, 59, 58, 55	Betaine	Amino acid	Gawron-Skarbek et al. (2023)
2	118	1.14	112, 77, 73, 72, 59, 57	Valine	Amino acid	Pekkoh et al. (2022)
3	163	2.06	163, 145, 135, 117, 107, 91, 89, 79, 77, 39, 45	Umbelliferone	Coumarin	Iwai et al. (2004)
4	287	6.47	287, 177, 95	2, 5, 7, 4′- tetra-Hydroxy isoflavone	Flavonoid	Ahmed M. et al. (2022)
5	391	6.69	149	Cinnamic acid derivative	Phenyl propanoid	Shoaib et al. (2017)
6	149	9.40	149, 148, 121, 107, 105, 103, 95, 93, 91, 81, 79, 77, 69, 67, 55, 43, 41	Cinnamic acid	Phenyl propanoid	Shoaib et al. (2017)
7	391	12.63	149	Cinnamic acid derivative	Phenyl propanoid	Shoaib et al. (2017)
8	167	12.73	167, 121, 106, 91, 85, 79, 77	Methoxy- phenyl acetic acid	Phenyl acetic acid	Ahmed M. et al. (2022), Murillo et al. (2019), Shoaib et al. (2017)
9	219	12.99	219, 203, 179, 175, 145, 143, 137, 133, 121, 119, 93, 92, 83, 81, 79, 69, 67, 43	Vulgarone b	Sesquiterpene	Ahmed M. et al. (2022), Murillo et al. (2019), Radi et al. (2023), Shoaib et al. (2017)
10	277	14.24	235, 217, 185, 171, 135, 123, 121, 119, 109, 107, 95, 93, 91, 83, 81, 79, 69, 67, 57, 55	Stearidonic acid	Fatty acid	Ahmed M. et al. (2022), Jeon et al. (2005), Murillo et al. (2019), Radi et al. (2023), Shoaib et al. (2017)
11	359	14.40	359, 84.3	11,12-Dimethoxy-8,11,13-abietatrien-20,7-olide	Diterpene	Ahmed M. et al. (2022), Jeon et al. (2005), Murillo et al. (2019), Radi et al. (2023), Shati et al. (2020), Shoaib et al. (2017), Sitthisuk et al. (2025)
12	197	15.32	151, 69	Dodecadienoic acid	Fatty acid	Ahmed M. et al. (2022), Jeon et al. (2005), Murillo et al. (2019), Radi et al. (2023), Shati et al. (2020), Shoaib et al. (2017), Sitthisuk et al. (2025), Zeng et al. (2015)
13	277	16.14	277, 235, 181, 153, 149, 135, 119, 107, 95	Caffeic acid derivative	Phenolic (Phenyl propanoid)	Kareem et al. (2025)
14	149	16.41	149, 121, 107, 93, 81, 65	cis-Cinnamic acid	Phenyl propanoid	Shoaib et al. (2017)
15	313	17.16	239, 189, 173,151, 123, 109, 103, 95, 85, 81, 75, 71, 69, 57	5-Hydroxy-7-eicosanone	Long-chain fatty alcohol	Ahmed M. et al. (2022), Chen et al. (2025), Jeon et al. (2005), Kareem et al. (2025), Murillo et al. (2019), Radi et al. (2023), Shati et al. (2020), Shoaib et al. (2017), Sitthisuk et al. (2025), Zeng et al. (2015)
16	167	18.51	99, 93, 85	Marmelolactone a	Monoterpene	Ahmed M. et al. (2022)
17	197	18.94	107, 99, 97, 95, 83, 81, 69, 56, 55, 43	(−)- Linalyl acetate	Monoterpene	Ahmed M. et al. (2022), Alotaibi et al. (2021), Chen et al. (2025), Jeon et al. (2005), Kareem et al. (2025), Murillo et al. (2019), Radi et al. (2023), Shati et al. (2020), Shoaib et al. (2017), Sitthisuk et al. (2025), Zeng et al. (2015)
18	279	19.24	279, 159, 135,132, 123, 121, 109, 105, 97, 95, 93, 83, 81, 67, 55	alpha Linolenic acid	Fatty acid	Ahmed M. et al. (2022), Alotaibi et al. (2021), Chen et al. (2025), Jeon et al. (2005), Kareem et al. (2025), Murillo et al. (2019), Radi et al. (2023), Shati et al. (2020), Shoaib et al. (2017), Sitthisuk et al. (2025), Zeng et al. (2015), Zhang et al. (2025)
19	313	20.77	123, 109, 103, 99, 97, 95, 89, 85, 83, 81, 71, 69, 67, 57, 55, 43	4-Hydroxy-6-eicosanone	Long-chain fatty alcohol	Ahmed M. et al. (2022), Chen et al. (2025), Jeon et al. (2005), Kareem et al. (2025), Murillo et al. (2019), Radi et al. (2023), Shati et al. (2020), Shoaib et al. (2017), Sitthisuk et al. (2025), Zeng et al. (2015)
20	313	22.47	111, 107, 85, 83, 71, 57, 56.7, 43	Eicosanoic acid	Fatty acid	Ahmed M. et al. (2022), Chen et al. (2025), Jeon et al. (2005), Kareem et al. (2025), Murillo et al. (2019), Radi et al. (2023), Shati et al. (2020), Shoaib et al. (2017), Sitthisuk et al. (2025), Zeng et al. (2015)
21	149	22.84	121, 93, 79, 69	Cuminaldehyde	Monoterpene	Ahmed M. et al. (2022), Alotaibi et al. (2021), Chen et al. (2025), Jeon et al. (2005), Kareem et al. (2025), Murillo et al. (2019), Radi et al. (2023), Shati et al. (2020), Shoaib et al. (2017), Sitthisuk et al. (2025), Zeng et al. (2015), Zhang et al. (2025)
22	341	23.60	111, 109, 99, 97, 95, 85, 83, 81, 71, 69, 67, 57, 55, 43	Docosanoic acid	Fatty acid	Ahmed M. et al. (2022), Alotaibi et al. (2021), Chen et al. (2025), Gong et al. (2020), Jeon et al. (2005), Kareem et al. (2025), Murillo et al. (2019), Radi et al. (2023), Shati et al. (2020), Shoaib et al. (2017), Sitthisuk et al. (2025), Zeng et al. (2015), Zhang et al. (2025)
23	569	24.12	243, 197, 159, 145, 119, 95, 79, 41	(3R,3′R,9Z)-Zeaxanthin	Tetraterpene	Ahmed M. et al. (2022), Alotaibi et al. (2021), Chen et al. (2025), Gong et al. (2020), Jeon et al. (2005), Kareem et al. (2025), Lin et al. (2016), Murillo et al. (2019), Radi et al. (2023), Shati et al. (2020), Shoaib et al. (2017), Sitthisuk et al. (2025), Zeng et al. (2015), Zhang et al. (2025)
24	414	25.23	396, 261, 137, 107	Spinasterol	Sterol	Salem et al. (2020)

**TABLE 2:** Secondary metabolites tentatively identified from the aerial parts extract of *Symphyotrichum squamatum* in negative ionization mode.

No.	M-H	Rt	MS^2^ (m/z)	Compound name	Class	References
1	353	0.77	191	5-Caffeoylquinic acid	Phenolic (Phenyl propanoid)	Ahmed M. et al. (2022), Alotaibi et al. (2021), Chen et al. (2025), Gong et al. (2020), Jeon et al. (2005), Kareem et al. (2025), Lin et al. (2016), Murillo et al. (2019), Radi et al. (2023), Salem et al. (2020), Shati et al. (2020), Shoaib et al. (2017), Sitthisuk et al. (2025), Sundaramoorthy and Packiam (2020), Zeng et al. (2015), Zhang et al. (2025)
2	275	2.90	155	Ginsenoyne c	Acetylenic (long-chain fatty alcohol)	Ahmed M. et al. (2022), Alotaibi et al. (2021), Chen et al. (2025), Gong et al. (2020), Jeon et al. (2005), Kareem et al. (2025), Lee et al. (2022), Lin et al. (2016), Murillo et al. (2019), Radi et al. (2023), Salem et al. (2020), Shati et al. (2020), Shoaib et al. (2017), Sitthisuk et al. (2025), Sundaramoorthy and Packiam (2020), Zeng et al. (2015), Zhang et al. (2025)
3	515	6.40	191, 179, 173, 135	4,5- Dicaffeoyl quinic acid	Phenolic (Phenyl propanoid)	Ahmed M. et al. (2022), Alotaibi et al. (2021), Chen et al. (2025), Gong et al. (2020), Jeon et al. (2005), Kareem et al. (2025), Lee et al. (2022), Lin et al. (2016), Mohanta et al. (2023), Murillo et al. (2019), Radi et al. (2023), Salem et al. (2020), Shati et al. (2020), Shoaib et al. (2017), Sitthisuk et al. (2025), Sundaramoorthy and Packiam (2020), Zeng et al. (2015), Zhang et al. (2025)
4	265	8.37	179	Malonyl caffeic acid derivative	Phenolic (Phenyl propanoid)	Kareem et al. (2025)
5	515	13.18	352,191, 81	1,4-Dicaffeoyl quinic acid	Phenolic (Phenyl propanoid)	Gong et al. (2020)
6	325	13.75	171	Protocatechuic acid derivative	Phenolic	Kim et al. (2020)
7	293	14.24	235, 191, 97, 71, 59	(2′e,4′z,8e)-Colneleic acid	Fatty acid	Ahmed M. et al. (2022), Alotaibi et al. (2021), Chen et al. (2025), Gong et al. (2020), Jeon et al. (2005), Kareem et al. (2025), Kim et al. (2020), Lee et al. (2022), Lin et al. (2016), Mohanta et al. (2023), Murillo et al. (2019), Radi et al. (2023), Salem et al. (2020), Shati et al. (2020), Shoaib et al. (2017), Sitthisuk et al. (2025), Sundaramoorthy and Packiam (2020), Zeng et al. (2015), Zhang et al. (2025)
8	279	15.32	97	3b-Hydroxy-6a-methoxy-7 (11)-eremophilen-12,8b-olide	Sesquiterpene	Ahmed M. et al. (2022), Alotaibi et al. (2021), Chen et al. (2025), Gong et al. (2020), Jeon et al. (2005), Kareem et al. (2025), Kim et al. (2020), Lee et al. (2022), Lin et al. (2016), Liu et al. (2025), Mohanta et al. (2023), Murillo et al. (2019), Radi et al. (2023), Salem et al. (2020), Shati et al. (2020), Shoaib et al. (2017), Sitthisuk et al. (2025), Sundaramoorthy and Packiam (2020), Zeng et al. (2015), Zhang et al. (2025)
9	295	16.70	295, 171, 59	12-Hydroxy-8,10-octadecadienoic acid	Fatty acid	Ahmed M. et al. (2022), Alotaibi et al. (2021), Chen et al. (2025), Gong et al. (2020), Jeon et al. (2005), Kareem et al. (2025), Kim et al. (2020), Lee et al. (2022), Lin et al. (2016), Liu et al. (2025), Mohanta et al. (2023), Murillo et al. (2019), Radi et al. (2023), Salem et al. (2020), Shati et al. (2020), Shoaib et al. (2017), Sitthisuk et al. (2025), Sundaramoorthy and Packiam (2020), Yang et al. (2022), Zeng et al. (2015), Zhang et al. (2025)
10	265	22.83	97	3-Methylgallic acid derivative	Phenolic	Ahmed M. et al. (2022)
11	515	25.23	515, 429	Ganoderenic acid c	Triterpene	Ahmed M. et al. (2022), Alotaibi et al. (2021), Chen et al. (2025), Gong et al. (2020), Habtemariam (2011), Jeon et al. (2005), Kareem et al. (2025), Kim et al. (2020), Lee et al. (2022), Lin et al. (2016), Liu et al. (2025), Mohanta et al. (2023), Murillo et al. (2019), Radi et al. (2023), Salem et al. (2020), Shati et al. (2020), Shoaib et al. (2017), Sitthisuk et al. (2025), Sundaramoorthy and Packiam (2020), Yang et al. (2022), Zeng et al. (2015), Zhang et al. (2025)
12	325	27.42	325	Heneicosanoic acid	Fatty acid	Ahmed M. et al. (2022), Alotaibi et al. (2021), Chen et al. (2025), Gong et al. (2020), Habtemariam (2011), Jeon et al. (2005), Kareem et al. (2025), Kim et al. (2020), Lee et al. (2022), Lin et al. (2016), Liu et al. (2025), Mohanta et al. (2023), Murillo et al. (2019), Radi et al. (2023), Ricciotti and FitzGerald (2011), Salem et al. (2020), Shati et al. (2020), Shoaib et al. (2017), Sitthisuk et al. (2025), Sundaramoorthy and Packiam (2020), Yang et al. (2022), Zeng et al. (2015), Zhang et al. (2025)

### 3.3 Total phenolic and total flavonoid contents of the hydro-alcoholic extract of *Symphyotrichum squamatum*

The Folin–Ciocalteu and Aluminum chloride methods for measuring total phenolics and total flavonoids showed that *S. squamatum* hydro-alcoholic extract is high in these compounds, with values of 65.9 ± 3.6 mg GAE/g extract for phenolics and 25.8 ± 1.1 mg quercetin equivalents per gram of extract for flavonoids.

### 3.4 Antioxidant activity of the hydro-alcoholic extract of *Symphyotrichum squamatum*

**FIGURE 4:**
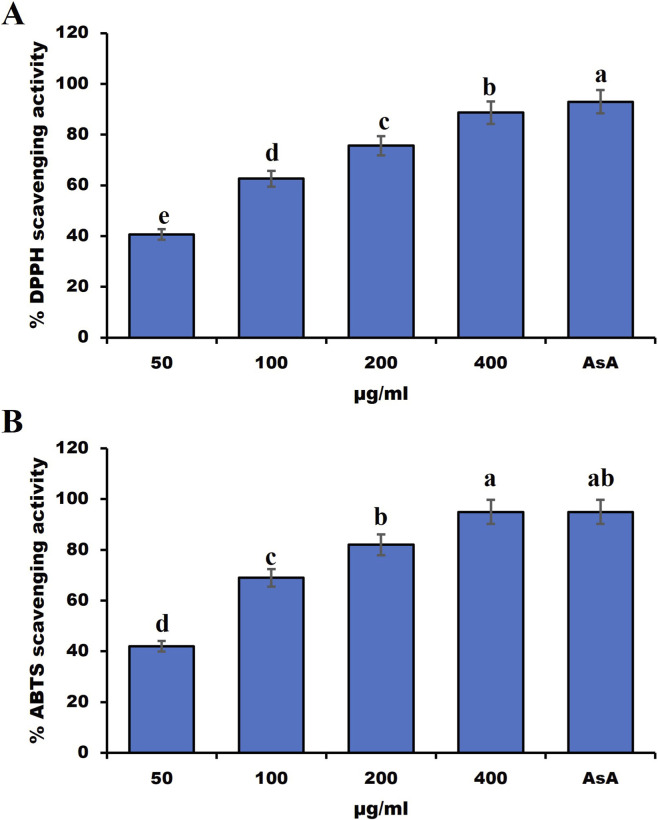
Antioxidant activity of hydro-alcoholic extract of aerial parts of *Symphyotrichum squamatum* against **(A)** DPPH and **(B)** ABTS free radicals. Lowercase letters (a–e) above columns indicate significant differences at *p* < 0.05.

*Symphyotrichum squamatum*’s hydro-alcoholic extract demonstrated significant antioxidant activity as measured by DPPH and ABTS methods, shown in Figures 4A,B. The hydro-alcoholic extract (400 μg/mL) scavenged 90% of DPPH radicals and 94.333% of ABTS radicals; these levels are very close to those achieved by ascorbic acid [48]. The SC50 values for DPPH and ABTS were 77.003 ± 2.745 μg/mL and 66.004 ± 2.317 μg/mL, respectively (Supplemental data; Supplementary Figures S2,S3).

### 3.5 Cytotoxic activity of the hydro-alcoholic extract of *Symphyotrichum squamatum*

**FIGURE 5:**
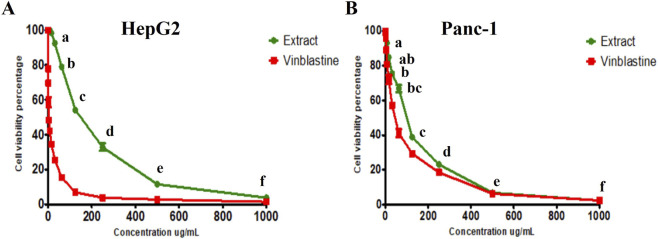
Cytotoxic activity of hydro-alcoholic extract of aerial parts of *Symphyotrichum squamatum* and vinblastine sulfate against **(A)** Hep-G2 cells and **(B)** Panc-1 cells. Lowercase letters (a–f) indicate significant differences between the extract and the positive control at *p* < 0.05.

The effect of the hydro-alcoholic extract on the growth of Hep-G2 and Panc-1 cells showed weak activity with marginal inhibition of cell proliferation, as indicated by their IC_50_ values of 149.57 ± 5.01 μg/mL and 100.04 ± 2.98 μg/mL, respectively. The common anticancer agent vinblastine sulfate had IC_50_ values of 3.69 ± 0.15 μg/mL and 41.84 ± 2.18 μg/mL for the 2 cell lines, respectively (Figure 5).

**FIGURE 6:**
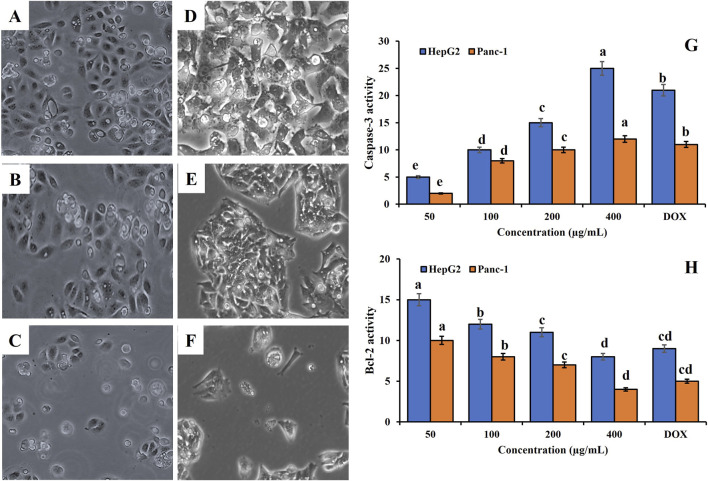
Evaluation of the pro-apoptotic effects of *Symphyotrichum squamatum* hydro-alcoholic extract on HepG2 and Panc-1 cancer cell lines. **(A–F)** Morphological Characterization: Phase-contrast micrographs showing untreated controls **(A,D)** cells treated with Doxorubicin **(B,E)** and cells treated with *S. squamatum* extract **(C,F)** for Panc-1 and HepG2 lines, respectively. **(G)** Caspase-3 Activity: Dose-dependent induction of executioner caspase activity following 24 h treatment. **(H)** Bcl-2 Activity: Dose-dependent downregulation of anti-apoptotic Bcl-2 expression, indicating mitochondrial pathway involvement. Lowercase letters (a–e) indicate significant differences between the extract and the positive controls at *p* < 0.05.

Figure 6 showed the mechanistic evaluation of the cytotoxic effects of *S. squamatum* (Aster) extract. The morphological analysis in Figures 6A–F showed that phase-contrast microscopy reveals that untreated Panc-1 (Figure 6A) and HepG2 (Figure 6D) cells maintained normal polygonal morphology and high confluency. The treatment with Doxorubicin (Figures 6B,E) and Aster extract (Figures 6C,F) induced significant apoptotic features, including cell shrinkage, cytoplasmic condensation, and a marked reduction in cell density.

Additionally, *Caspase*-3 Activation (Figure 6G): The extract induced a significant, dose-dependent increase in *Caspase*-3 activity. In HepG2 cells, the activity at 400 μg/mL was approximately 5-fold higher than the basal level, significantly outperforming the positive control, Doxorubicin (DOX). Also, Bcl-2 Downregulation (Figure 6H), consistent with the induction of apoptosis, the extract treatment resulted in a dose-dependent reduction in *Bcl*-2 activity. This suppression of anti-apoptotic signaling was most pronounced at 400 μg/mL, confirming that the extract interferes with the mitochondrial pathway of programmed cell death.

### 3.6 Antidiabetic activity

#### 3.6.1 α-glucosidase inhibition assay

**FIGURE 7:**
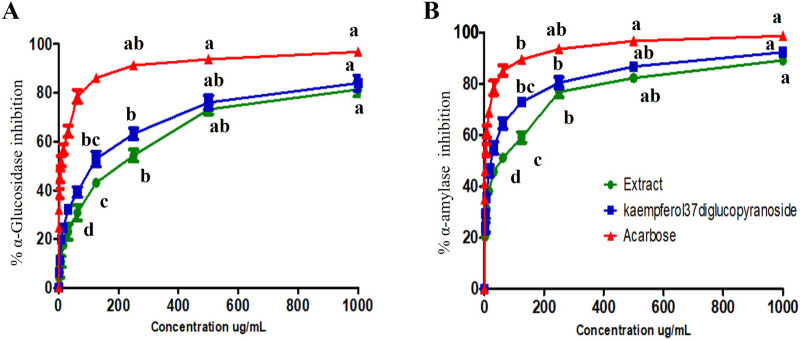
Inhibition percentage of **(A)** α-Glucosidase and **(B)** α-amylase by *Symphyotrichum squamatum* aerial part extract and kaempferol-3,7-diglucopyranoside compared to the acarbose standard. Lowercase letters (a-d) indicate significant differences between the extract and the positive controls at *p* < 0.05.

The ability of the plant extract (A) and kaempferol-3,7-diglucopyranoside (K) to inhibit α-glucosidase enzyme activity was assessed at various concentrations ranging from 2 to 1000 μg/mL. The tested samples exhibited α-glucosidase inhibition in a dose-dependent manner, as shown in Figure 7. Maximum inhibition levels of 81.35%, 83.96%, and 96.74% were observed at 1000 μg/mL for (A), (K), and the acarbose standard, respectively. The IC50 values were 202.42 ± 6.24, 111.89 ± 3.06, and 6.59 ± 0.35 μg/mL for (A), (K), and acarbose, respectively.

#### 3.6.2 α-amylase inhibition assay

**FIGURE 8:**
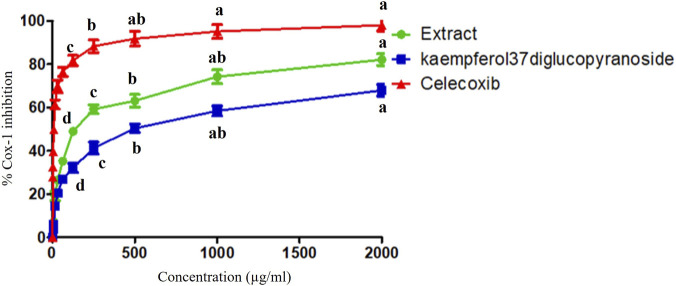
Inhibition percentage of COX-1 by the aerial part extract of *Symphyotrichum squamatum* and kaempferol-3,7-diglucopyranoside compared to the Celecoxib standard. Lowercase letters (a–d) indicate significant differences between the extract and the positive controls at *p* < 0.05.

Inhibition of α-amylase activity by the plant extract (A), kaempferol-3,7-diglucopyranoside (K), and acarbose was found to be dose-dependent from 2 to 1000 μg/mL concentrations, as shown in Figure 8. Maximum inhibition of 89.21%, 92.34%, and 98.69% was observed at 1000 μg/mL for (A), (K), and acarbose, respectively. The IC50 values were 55.48 ± 1.64, 24.29 ± 0.75, and 2.97 ± 0.15 μg/mL for (A), (K), and acarbose, respectively.

### 3.7 Anti-inflammatory activity (COX-1 inhibition assay)

The tested extracts (A) and kaempferol-3,7-diglucopyranoside (K) were examined for their ability to inhibit human cyclooxygenase (COX-1) activity, compared with celecoxib, a standard drug. The results are shown in Figure 7. The COX enzyme was inhibited by the tested extract in a dose-dependent manner. The extract (A) showed significant potency against COX-1, with inhibition reaching 82.05% at 2000 μg/mL and an IC_50_ of 137.51 ± 3.47 μg/mL. Additionally, kaempferol-3,7-diglucopyranoside inhibited COX-1 by 67.91% at 2000 μg/mL, with an IC_50_ of 490.36 ± 10.22 μg/mL. In contrast, the IC_50_ of the standard drug, celecoxib, for COX-1 was 7.94 ± 0.32 μg/mL. The extract showed a result superior to other known plants like *Acacia catechu* and *Euphorbia helioscopia* that showed similar percentage of inhibition of 79.96% and 85.68% but at a higher dose of 3,400 μg/mL. Therefore, the tested plant extract could be a promising anti-inflammatory agent.

### 3.8 Molecular docking of components from *Symphyotrichum squamatum’s* aerial parts with α-glucosidase and α-amylase enzymes

The extract of *S. squamatum*’s aerial parts was found to have an anti-diabetic effect by inhibiting β-glucosidase, α-amylase, or both simultaneously. To understand how the main components in the extract bind to these enzymes, a molecular docking tool was evaluated.

The docked compounds exhibited docking scores ranging from −3.8700 to −8.0053 kcal/mol for α-glucosidase and from −4.0155 to −9.1527 kcal/mol for α-amylase, as shown in Table 3.

**TABLE 3:** Docking scores for the major constituents in *Symphyotrichum squamatum* aerial parts extract when docked into α-glucosidase (PDB ID: 3W37) and α-amylase (PDB ID: 3BAJ).

No.	Compound	Docking score (kcal/mol), Rmsd (Å)	
		α-glucosidase	α-amylase
1	Methoxy- phenyl acetic acid	−4.9843, 0.6899	−4.6609, 0.9415
2	Vulgarone b	−4.3259, 1.4825	−5.0340, 0.8120
3	Stearidonic acid	−5.5994, 1.3026	−6.0756, 1.3045
4	11,12-Dimethoxy-8,11,13-abietatrien-20,7-olide	−5.6254, 1.3622	−5.9807, 1.3184
5	Dodecadienoic acid	−5.1609, 1.2687	−5.4211, 1.5443
6	cis-Cinnamic acid	−4.3035, 0.9940	−4.5239, 1.6278
7	Marmelolactone a	−4.7575, 1.2095	−4.9873, 0.7227
8	(−)- Linalyl acetate	−5.4613, 1.4500	−5.2295, 0.9952
9	alpha Linolenic acid	−5.8462, 1.0536	−6.3948, 1.2506
10	4-Hydroxy-6-eicosanone	−6.4907, 1.1135	−6.6669, 1.7417
11	Eicosanoic acid	−5.7989, 1.9384	−6.7152, 1.2064
12	Cuminaldehyde	−4.5642, 1.7839	−4.4009, 1.1393
13	Docosanoic acid	−6.1946, 1.5102	−7.0233, 1.2223
14	(3R,3′R,9Z)-Zeaxanthin	−5.8771, 2.2791	−7.6042, 1.5870
15	Spinasterol	−6.2185, 1.6421	−6.6587, 1.6776
16	(2′e,4′z,8e)-Colneleic acid	−5.5702, 1.3517	−6.9304, 1.3121
17	3b-Hydroxy-6a-methoxy-7 (11)-eremophilen-12,8b-olide	−4.8814, 0.8113	−6.0144, 1.4715
18	12-Hydroxy-8,10-octadecadienoic acid	−5.7909, 1.9962	−6.1065, 1.8159
19	Ganoderenic acid c	−6.5228, 1.8722	−7.6069, 1.2583
20	Heneicosanoic acid	−5.9395, 1.9855	−7.2985, 1.4681
21	Dioctyl phthalate	−6.9154, 1.7489	−7.2588, 1.3890
22	Friedelinol	−5.3197, 1.0151	−5.7234, 1.8646
23	Kaempferol-3,7-diglucopyraniside	−8.0053, 1.6244	−9.1527, 1.8134
24	Betaine	−4.2751, 1.2959	−4.0155, 1.1133
25	Valine	−4.1210, 0.8882	−4.0829, 1.8896
26	Umbelliferone	−3.9245, 1.4775	−4.3131, 0.7578
27	2, 5, 7, 4′- tetra-Hydroxy isoflavone	−4.6503, 1.9366	−5.4549, 1.1998
28	Cinnamic acid	−3.8700, 0.4289	−4.4327, 1.0194
29	5-Caffeoylquinic acid	−6.0989, 1.6001	−6.2638, 1.4433
30	Ginsenoyne c	−6.3146, 1.0464	−6.4527, 1.9104
31	4,5- Dicaffeoylquinic acid	−7.4641, 1.3002	−6.9206, 1.2272
32	1,4-Dicaffeoylquinic acid	−7.4572, 1.5458	−7.9011, 1.4030
33	Protocatechuic acid	−4.0982, 1.9312	−4.6259, 1.5209
​	References/co-crystalized structure	−9.6348, 1.7502	−12.5566, 1.7918

**FIGURE 9:**
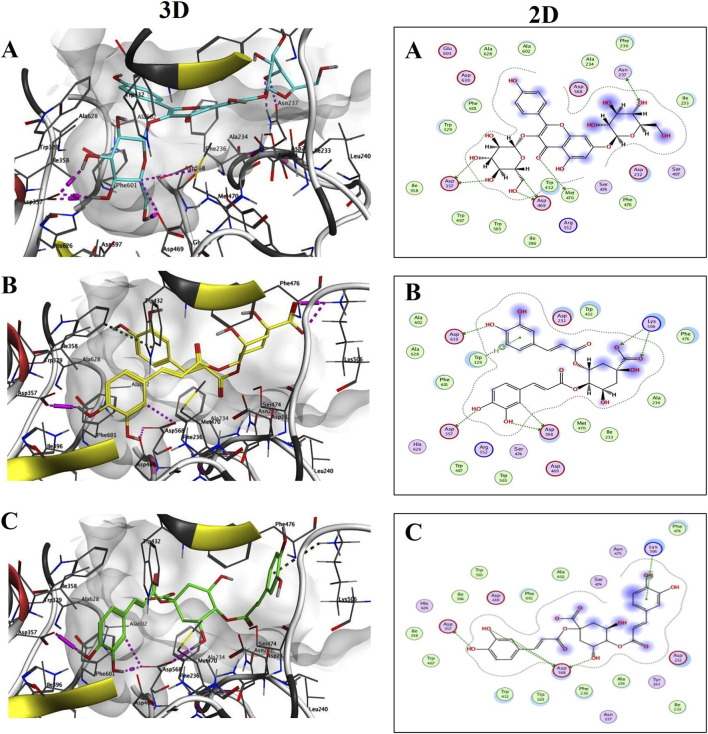
3D and 2D binding interactions of Kaempferol-3,7-diglucopyranoside; **(A)** (S = −8.0053), 4,5-Dicaffeoylquinic acid; **(B)** (S = −7.4641), and 1,4-Dicaffeoylquinic acid; **(C)** (S = −7.4572) at the α-glucosidase (PDB ID: 3W37) binding site.

α-glucosidase (PDB ID: 3W37) binds with kaempferol-3,7-diglucopyranoside (Figure 9A), 4,5-dicaffeoylquinic acid (Figure 9B), and 1,4-dicaffeoylquinic acid 1 (Figure 9C), demonstrating good fit with high binding scores. Kaempferol-3,7-diglucopyranoside forms seven hydrogen bonds with Asp357, Asp469, Asn237, and Met470. In contrast, 4,5-dicaffeoylquinic acid exhibits H-π interactions with Trp329 and five hydrogen bonds with Asp357, Asp568, and Lys506. Similarly, 1,4-dicaffeoylquinic acid forms four hydrogen bonds with Asp357 and Asp568, along with H-π interactions with Lys506 (Figure 8).

**FIGURE 10:**
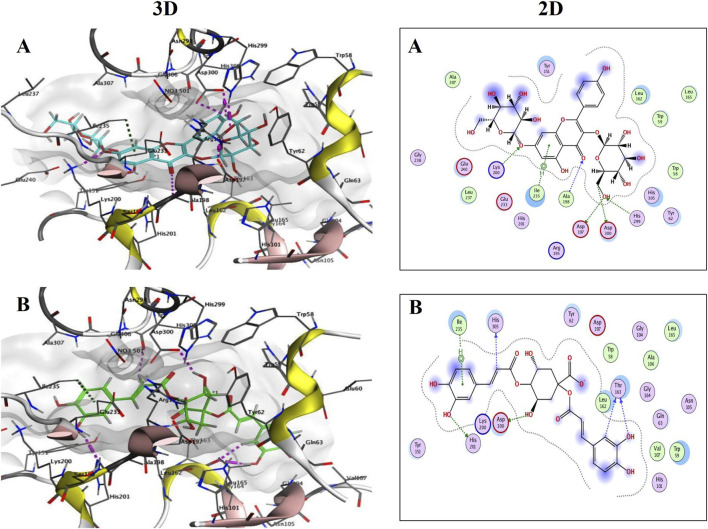
3D and 2D binding interactions of Kaempferol-3,7-diglucopyranoside; **(A)** (S = −9.1527) and 1,4-Dicaffeoylquinic acid; **(B)** (S = −7.9011) at the α-amylase (PDB ID: 3BAJ) active site.

Kaempferol-3,7-diglucopyranoside (Figure 10A) and 1,4-dicaffeoylquinic acid (Figure 10B) showed high binding scores with interesting interactions at the α-amylase (PDB ID: 3BAJ) active site. Kaempferol-3,7-diglucopyranoside revealed 5 HBs with ASP197, Ala198, Lys200, His299 and Asp300 besides H-π interactions with Ile235. Whereas, the 1,4-dicaffeoylquinic acid also formed 5 HBs with Thr163, His201, Asp300 and His 305 in addition to H-π interactions with Ile235. Interestingly, the docking results for kaempferol-3,7-diglucopyranoside showed binding interactions with both α-glucosidase and α-amylase, which help elucidate the possible mechanisms underlying the antidiabetic activity of the *S. squamatum* aerial parts extract.

### 3.9 Docking studies at COX-1 and COX-2 enzymes

The molecular docking studies provide a detailed evaluation of how the major constituents of *Symphyotrichum squamatum* interact with cyclooxygenase enzymes, which are primary targets for anti-inflammatory therapy. The docking scores and RMSD values, as summarized in Table 4, reveal that the majority of identified compounds successfully bind to the active sites of both COX-1 and COX-2.

**TABLE 4:** Docking scores obtained upon docking the major constituents in *Symphyotrichum squamatum* aerial parts extract into COX-1 (PDB ID: 1EQG) and COX-2 (PDB ID: 1CX2).

No	Compound	Docking score (kcal/mol), Rmsd (Å)	
		COX-1	COX-2
1	Methoxy- phenyl acetic acid	−5.4246, 1.5472	−5.3097, 1.6183
2	Vulgarone b	−2.5181, 1.3247	−3.3635, 1.5838
3	Stearidonic acid	−7.3529, 1.2454	−8.1308, 1.2832
4	11,12-Dimethoxy-8,11,13-abietatrien-20,7-olide	−2.3079, 1.8399	−3.5807, 1.2676
5	Dodecadienoic acid	−6.5197, 0.8957	−7.3639, 1.6873
6	cis-Cinnamic acid	−5.0962, 1.5572	−5.3256, 0.7733
7	Marmelolactone a	−5.6354, 1.2502	−6.0883, 0.7904
8	(−)- Linalyl acetate	−6.1449, 1.6559	−6.5542, 1.3650
9	alpha Linolenic acid	−7.4989, 1.6086	−8.0291, 1.2666
10	4-Hydroxy-6-eicosanone	−8.0431, 1.1143	−7.4140, 1.4784
11	Eicosanoic acid	−8.2811, 1.4495	−8.4872, 1.6468
12	Cuminaldehyde	−5.3807, 1.2981	−5.6350, 1.0654
13	Docosanoic acid	−8.3080, 1.9675	−5.7971, 1.3164
14	(3R,3′R,9Z)-Zeaxanthin	failed	failed
15	Spinasterol	−3.1012, 1.5385	failed
16	(2′e,4′z,8e)-Colneleic acid	−7.6833, 1.9070	−8.0315, 1.4875
17	3b-Hydroxy-6a-methoxy-7 (11)-eremophilen-12,8b-olide	−2.7994, 0.8799	−4.0337, 1.5911
18	12-Hydroxy-8,10-octadecadienoic acid	−8.0284, 1.6593	−6.8307, 1.5342
19	Ganoderenic acid c	−1.0740, 1.6747	failed
20	Heneicosanoic acid	−8.5837, 1.8203	−6.0157, 1.4982
21	Dioctyl phthalate	−8.4732, 1.5144	−6.8044, 1.5763
22	Friedelinol	failed	failed
23	Kaempferol-3,7-diglucopyraniside	−7.9055, 1.6350	−4.9487, 2.0170
24	Betaine	−4.5758, 0.9259	−4.6265, 1.2830
25	Valine	−4.6409, 0.6600	−4.7694, 1.5865
26	Umbelliferone	−4.9542, 0.8921	−5.2469, 1.7179
27	2, 5, 7, 4′- tetra-Hydroxy isoflavone	−7.2864, 0.8240	−6.7146, 1.2491
28	Cinnamic acid	−4.9215, 1.2221	−5.3539, 1.5884
29	5-Caffeoylquinic acid	−6.2818, 1.8268	−7.5915, 1.1344
30	Ginsenoyne c	−7.8073, 1.9450	−8.5450, 1.7836
31	4,5- Dicaffeoylquinic acid	−4.0329, 1.3027	−3.1754, 1.8982
32	1,4-Dicaffeoylquinic acid	−8.3742, 1.4121	−5.6623, 1.5264
33	Protocatechuic acid	−4.9168, 0.9799	−5.1438, 1.9351
​	References/co-crystalized structure	−7.2574, 0.8973	−10.0818, 1.1237

**FIGURE 11:**
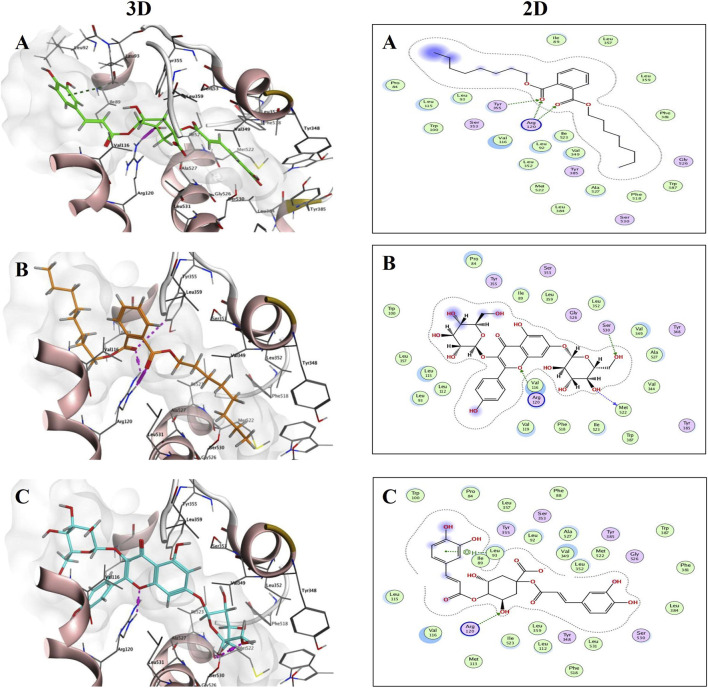
3D and 2Dbinding interactions of Dioctyl phthalate; **(A)** (S = −8.4732), Kaempferol-3,7-diglucopyraniside; **(B)** (S = −7.9055) and 1,4-Dicaffeoylquinic acid; **(C)** (S = −8.3742) at COX-1 (PDB ID: 1EQG) binding site.

For the COX-1 enzyme (PDB ID: 1EQG), the binding scores ranged from −2.5181 to −8.4732. Notably, compounds such as Dioctyl phthalate (S = −8.4732), 1,4-Dicaffeoylquinic acid (S = −8.3742), and kaempferol-3,7-diglucopyranoside (S = −7.9055) outperformed the re-docked crystallized ibuprofen, which had a binding score of −7.2574. Specific molecular interactions support the high affinity of these compounds: Dioctyl phthalate established three hydrogen bonds with Tyr355 and Arg120, and kaempferol-3,7-diglucopyranoside formed two hydrogen bonds with Arg120 and Met522. As shown in Figure 11, these 3D and 2D interactions illustrate the spatial orientation of the ligands within the protein’s binding pocket. Furthermore, Figure 11 highlights that 1,4-Dicaffeoylquinic acid stabilized its position through a hydrogen bond with Arg120 and H-π interactions with Leu93.

**FIGURE 12:**
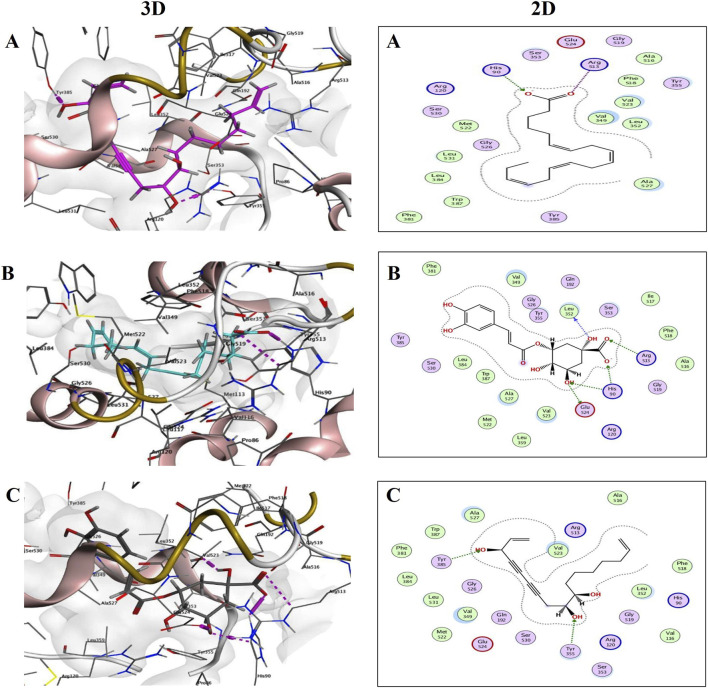
3D and 2D binding interactions of Stearidonic acid; **(A)** (S = −8.1308), 5-Caffeoylquinic acid; **(B)** (S = −7.5915) and Ginsenoyne c; **(C)** (S = −8.5450) at COX-2 (PDB ID: 1CX2) binding site.

In the case of the COX-2 enzyme (PDB ID: 1CX2), binding scores ranged from −3.3635 to −8.5450. Figure 12 provides a detailed visualization of the top-performing compounds in this category. Stearidonic acid (S = −8.1308) was found to form two hydrogen bonds with Arg513 and His90 (Figure 12A), while 5-caffeoylquinic acid (S = −7.5915) demonstrated a more extensive network with five hydrogen bonds involving Arg513, His90, Glu524, and Leu352 (Figure 12B). Ginsenoyne C achieved the highest score for COX-2 at −8.5450, forming hydrogen bonds with Tyr355 and Tyr385 (Figure 12C).

The interpretation of these results indicates varying degrees of enzyme selectivity among the constituents. For instance, 1,4-Dicaffeoylquinic acid demonstrated higher selectivity for COX-1, whereas eicosanoic acid showed significant binding affinity for both COX-1 (S = −8.2811) and COX-2 (S = −8.4872). This suggests that the aerial parts of *S. squamatum* contain a complex mixture of compounds that may provide a multi-targeted approach to inhibiting inflammatory pathways.

## 4 Discussion

The Asteraceae family is rich in phytochemicals known for their biological significance and beneficial effects on human health (Rolnik and Olas, 2021). Reactive oxygen species are a major cause of various human diseases, including neurodegenerative disorders, cardiovascular conditions, and diabetes (Gonzalez-Burgos et al., 2012). *Symphyotrichum squamatum* is one of the aster species with limited information on its chemical makeup and biological activities. In this study, analysis of the plant’s hydroalcoholic extract using HPLC-PDA-ESI-MS/MS identified 35 secondary metabolites; additionally, column chromatography of the plant’s petroleum ether and ethyl acetate fractions separated four compounds, including spinasterol, identified by HPLC-MS/MS.

The antioxidant activity of the extract was confirmed using DPPH and ABTS methods. Pathway analysis of the identified compounds showed that, on one hand, fatty acids, phenolic propanoids, and phenyl propanoids are the main metabolic components of the plant. Recent studies have confirmed the potential role of fatty acids as antioxidants and their protective effects against cardiovascular disorders by increasing plasma and salivary total antioxidant capacity and inhibiting angiotensin-converting enzyme (Gawron-Skarbek et al., 2023; Pekkoh et al., 2022). The high levels of these polyphenolic compounds in the extract explain the antioxidant activity of the *S. squamatum* hydro-alcoholic extract. Similar effects were shown in Korkina (2007), who found that bioactive compounds extracted from *Caralluma edulis*—particularly in methanol—demonstrate significant antioxidant and anti-inflammatory activities, as well as antidiabetic potential in subacute animal models. This scientific validation supports the plant’s traditional use and its role in managing metabolic biomarkers through phytochemicals like phenolics and flavonoids. Phenyl propanoids and phenolic propanoids are also powerful antioxidants, and their capacity could support wound healing, as well as anti-inflammatory, anticancer, antibacterial, and antiviral activities (Korkina, 2007). 4,5-Dicaffeoylquinic acid was identified as the major constituent in the negative ionization mode chromatogram, along with other caffeoyl quinic acids. These compounds are classified as phenolic propanoids with high antioxidant potential, comparable to common antioxidants such as ascorbic acid and alpha-tocopherol (Iwai et al., 2004). Additionally, several terpenes, including sesquiterpenes, monoterpenes, diterpenes, sterols, triterpenes, and tetraterpenes, were identified in the extract. Terpenes constitute another class of antioxidant compounds (Gonzalez-Burgos et al., 2012), further supporting the extract’s antioxidant activity. Spinasterol was identified as the major compound in the positive-ion chromatogram and isolated from the petroleum ether fraction. Research by Ahmed et al. indicated its significant antioxidant activity as well as antifungal and aphicidal properties (Ahmed M. et al., 2022). Moreover, a previous study on the sesquiterpene components of *Artemisia macrocephala* (a plant of the Asteraceae Family) reported promising antioxidant, antitumor, and mild-to-moderate antinociceptive activities (Shoaib et al., 2017). Interestingly, the tetraterpene zeaxanthin was also detected in the extract. As a plant carotenoid (provitamin A), it is known for its potential antioxidant activity (Murillo et al., 2019). Since zeaxanthin is fat-soluble, its bioavailability is enhanced when ingested with fatty matter. Therefore, the coexistence of fatty acids and fatty alcohols with zeaxanthin in the extract could amplify its antioxidant effect.

Regarding cytotoxic activity, the hydroalcoholic extract exhibited weak effects on the proliferation of Hep-G2 and Panc-1 cells. This activity may result from some of its phytoconstituents. For example, friedelinol, isolated from the petroleum ether fraction, might contribute to this cytotoxic effect. The beta form of this compound has been shown to exhibit cytotoxicity, as well as antibacterial and antiviral effects (Radi et al., 2023). Spinasterol, another compound that was isolated in this study was found to exhibit cytotoxicity against various cancer cell lines, including MCF-7, ZR-75–1, MDA-MB-231, SK-BR-3, and Hs578T (Jeon et al., 2005); it may be one of the components responsible for the extract’s cytotoxicity. Other components in the extract could mask the activity of the aforementioned compounds. Further research is still needed to confirm the component(s) responsible for *S. squamatum*’s cytotoxic effects.

The description of Figure 6 indicates that the Aster (*S. squamatum*) extract exerts cytotoxicity in Panc1 and HepG2 cells through mitochondria-mediated apoptosis, supported at three mechanistic levels: morphology, caspase-3 activation, and Bcl-2 downregulation.

Phase-contrast microscopy shows that untreated Panc-1 and HepG2 cells retain normal polygonal shape and high confluency, whereas both Doxorubicin and Aster extract cause cell shrinkage, cytoplasmic condensation and reduced cell density. These are classical hallmarks of apoptosis rather than necrosis and are widely reported for cytotoxic plant extracts and natural compounds in cancer cells, including HepG2 and other epithelial lines (Sitthisuk et al., 2025). Nuclear condensation, fragmentation and apoptotic body formation under fluorescence microscopy have similarly been used to confirm apoptosis induced by *Saussurea costus, Mammea siamensis* and *Scutellaria barbata* extracts (Shati et al., 2020). Thus, the morphological changes in Figures 6A–F are consistent with programmed cell death.

Figure 6G shows a dose-dependent increase in caspase-3 activity, reaching ∼5-fold over basal in HepG2 at 400 μg/mL and exceeding Doxorubicin. Activation of caspase-3 is a central event in both extrinsic and intrinsic apoptotic pathways and is a common mechanistic endpoint for plant-derived anticancer agents (Shati et al., 2020; Sitthisuk et al., 2025). For example, sasanquasaponin in HepG2 cells upregulates caspase-3 at the mRNA and protein levels and drives irreversible apoptosis via the mitochondrial pathway (Zeng et al., 2015). *Saussurea costus* leaf extract similarly induces robust caspase-3 activation across HepG2, HCT116, and MCF-7 cells in parallel with growth inhibition (Kareem et al., 2025; Shati et al., 2020). *Scutellaria barbata* and its oils also activate caspase-3 (often together with caspase-9 and −8) during hepatoma cell apoptosis (Chen et al., 2025). The strong, dose-responsive caspase-3 activation by *S. squamatum* extract therefore fits well within an established mechanistic pattern for Asteraceae and other medicinal plants.

Figure 6H shows dose-dependent suppression of Bcl-2, particularly at 400 μg/mL. Bcl-2 is a key anti-apoptotic protein that stabilizes mitochondrial membranes and opposes Bax/Bak-mediated cytochrome c release. Numerous plant extracts that trigger intrinsic apoptosis exhibit the same signature: decreased Bcl-2, increased Bax (or Bax/Bcl-2 ratio), mitochondrial depolarization, cytochrome c release, and subsequent caspase-9/3 activation (Alotaibi et al., 2021; Zhang et al., 2025). For instance:

Sasanquasaponin in HepG2 lowers Bcl-2 while increasing Bax and caspase-3, indicating mitochondrial-dependent apoptosis (Zeng et al., 2015). Also, *Saussurea lappa* extract in HepG2 promotes cytochrome c release, upregulates Bax and caspase-3, and downregulates Bcl-2 (Alotaibi et al., 2021). Saussurea costus leaf fractions decrease *Bcl*-2 and elevate Bax and caspase-3/7 across liver, colon, and breast cancer cells. Additionally, fermented stevia-derived chlorogenic acid methyl ester in Panc-1 cells increases *Bax* and cytochrome c and decreases *Bcl*-2, together with caspase-9/3 upregulation (Rolnik and Olas, 2021).

These convergent findings support the interpretation that *S. squamatum* extract interferes with mitochondrial integrity, shifts the Bax/Bcl-2 balance toward apoptosis, and activates the intrinsic caspase cascade, with caspase-3 as the main executioner (Shati et al., 2020; Zeng et al., 2015).

Several Asteraceae members (Saussurea lappa, Saussurea costus, Centaurea species) exhibit potent cytotoxicity via Bcl-2 modulation and caspase-3-dependent intrinsic apoptosis in diverse cancer models (Shati et al., 2020). The pattern observed in Figure 6—apoptotic morphology, strong caspase-3 activation, and Bcl-2 suppression in Panc-1 and HepG2—aligns closely with this family-wide mechanistic profile and supports *S. squamatum* (Aster) as another promising mitochondrial-targeting phytotherapeutic candidate.

The promising antioxidant activity of the extract motivated us to explore its antidiabetic and anti-inflammatory effects. Regarding antidiabetic activity, α-glucosidase and α-amylase enzymes are essential for carbohydrate digestion, and controlling them is a common strategy to manage postprandial hyperglycemia (Gong et al., 2020). The hydro-alcoholic extract inhibited both enzymes. This activity could be due to its phenolic, flavonoid, and terpene contents (Lin et al., 2016; Salem et al., 2020). Interestingly, Kaempferol-3,7-diglucopyranoside, isolated from the ethyl acetate fraction, showed anti-α-glucosidase and α-amylase activity (Sundaramoorthy and Packiam, 2020). Additionally, 1,4-Dicaffeoylquinic acid, identified by LC-MS/MS, demonstrated antidiabetic effects by inhibiting α-glucosidase and stimulating glucose-induced insulin secretion (Lee et al., 2022). Molecular docking studies of digestive enzymes supported these results.

The potent, dose-dependent inhibition of α-glucosidase by A and K aligns with prior reports on Aster species and kaempferol derivatives (Mohanta et al., 2023). Aqueous and ethanolic extracts of *Aster koraiensis* leaves showed strong α-glucosidase inhibition with IC50 values in the mg/ml range and mixed-type kinetics, together with significant antioxidant capacity (Kim et al., 2020). Similarly, *Aster* (*Callistephus chinensis*) flower waste extract exhibited *in vitro* antidiabetic potential through carbohydrate-hydrolyzing enzyme inhibition and enhanced glucose uptake. Regarding α-amylase inhibitory effect of aster extract, some previously studied plant extracts agree with the current study like *Ampelopsis grossedentata* with IC_50_ of 105.5 μg/mL, *Senna auriculata* with IC_50_ of 49.45 μg/mL, and brown rice with IC_50_ of 48.96 μg/mL (Awuchi and Morya, 2023; Liu et al., 2025). At the compound level, kaempferol has been characterized as a mixed-type α-glucosidase inhibitor with micromolar IC50 values and high binding affinity to the enzyme active site, while kaempferol-3,7-di-O-β-glucoside and related glycosides display strong inhibitory activity against both α-amylase and α-glucosidase, in some cases surpassing acarbose (Habtemariam, 2011; Yang et al., 2022). These findings support that the relatively low IC50 of K compared with the crude Aster extract in the present study is consistent with the recognized role of kaempferol glycosides as key antidiabetic constituents mediating enzyme inhibition.

The docking analysis provides clear insights into how compounds from the aerial parts of *S. squamatum* exert their antidiabetic and anti-inflammatory effects. The observed binding energies and ligand–protein interactions indicate a stable, favorable fit of these molecules within the active sites of α-glucosidase and α-amylase. These main enzymes control the breakdown of carbohydrates.

Regarding the anti-inflammatory activity, COX-1 and COX-2 enzymes catalyze the biosynthesis of prostaglandins, which trigger the inflammatory response (Ricciotti and FitzGerald, 2011; Rouzer and Marnett, 2009). The strong COX-1 inhibition produced by the Aster extract and its kaempferol glycoside is consistent with the recognized anti-inflammatory potential of Asteraceae species and kaempferol derivatives, and supports the positioning of this extract as a promising plant-derived anti-inflammatory candidate (Alam et al., 2020; Chagas-Paula et al., 2015).​

The marked inhibition of COX-1 by the Aster extract (82.05% at 2000 μg/mL; IC50 137.51 μg/mL) agrees with reports that many Asteraceae members exert anti-inflammatory effects via cyclooxygenase pathway modulation. A screening of selected Asteraceae species demonstrated potent dual inhibition of COX-1 and 5-lipoxygenase, with several extracts showing IC50 values close to or even below reference NSAID inhibitors, highlighting this family as a rich source of COX-targeting anti-inflammatory agents (Chagas-Paula et al., 2015).​

Beyond direct enzyme inhibition, methanolic extracts of *Aster incisus* significantly reduced COX-2 and iNOS expression and pro-inflammatory cytokines in LPS-stimulated cells, indicating that Aster constituents can downregulate prostaglandin biosynthesis and inflammatory signaling at both enzymatic and transcriptional levels (Ngabire et al., 2018). Similarly, ethanol extract of *Aster scaber* suppressed iNOS and COX-related mediators and reduced IL-1β and IL-6 in macrophages, reinforcing that Aster species display robust anti-inflammatory activity mediated through modulation of COX isoforms and downstream cytokines (Saba et al., 2022).​

The moderate COX-1 inhibition observed for kaempferol-3,7-diglucopyranoside (67.91% at 2000 μg/mL; IC50 490.36 μg/mL) is in line with growing evidence that kaempferol glycosides act as multi-target anti-inflammatory agents rather than ultra-potent COX inhibitors *per se* (Lee et al., 2018). Kaempferol 7-O-β-D-glucoside significantly downregulated iNOS and COX-2 protein and mRNA levels, as well as TNF-α, IL-1β, and IL-6 in LPS-stimulated RAW 264.7 macrophages by blocking NF-κB, AP-1 and JAK–STAT signaling, indicating potent suppression of prostaglandin-generating machinery and inflammatory gene expression (Alam et al., 2020).​

More recently, kaempferol 3-O-glucoside (K3G) was shown to inhibit COX-2 and iNOS expression and reduce NO and prostaglandin E synthase 2 in activated microglial cells, with effects linked to inhibition of MAPKs and NF-κB and activation of Nrf2/HO-1 (Lim et al., 2023). Comprehensive reviews conclude that kaempferol and its glycosides inhibit cyclooxygenase enzymes and lipooxygenase, and globally attenuate inflammatory mediator production, which supports the contribution of kaempferol-3,7-diglucopyranoside to the COX-1–directed activity observed in the present extract (Mahat et al., 2010).​

The Aster extract showed COX-1 inhibition comparable in magnitude, but at lower concentrations, than some other medicinal plants previously reported as COX-1 inhibitors, supporting its potent pharmacological profile. For example, chloroform and methanolic extracts of *Guettarda speciosa* displayed COX-1 IC50 values of 3.56 and 4.98 μg/mL with >60% inhibition at 10 μg/mL, illustrating that certain species can reach very high potency but are taxonomically unrelated to Aster (Kumari et al., 2022). In contrast, some Acacia and Euphorbia species, although well documented for anti-inflammatory, antioxidant and antidiabetic activities, generally require higher doses *in vivo* or show moderate percentage inhibition in enzyme assays, indicating that their effective concentrations are often higher than those used for standard NSAIDs (Batiha et al., 2022; Kumari et al., 2022; Rojas-Jiménez et al., 2024).​

Within Asteraceae, screening data revealed several species whose COX-1 inhibitory IC50 values approximate synthetic standards, and that dual COX-1/5-LOX inhibition is a characteristic feature, which is consistent with the high inhibition percentage (82.05%) obtained for the present Aster extract. These findings collectively suggest that the observed COX-1 inhibition by the Aster extract and its kaempferol glycoside is not an isolated phenomenon but fits within a broader pattern of strong cyclooxygenase modulation by Asteraceae plants and kaempferol-based flavonoids (Alam et al., 2020; Chagas-Paula et al., 2015).​

The lower IC50 of the crude extract in comparison to celecoxib (7.94 μg/mL) is anticipated, given that standard NSAIDs are optimized small molecules, whereas plant extracts are complex mixtures in which inert components dilute active constituents (Saba et al., 2022). Nevertheless, the significant inhibition observed at the tested doses, along with corroborating literature on Aster species and kaempferol glycosides, supports the notion that this extract could serve as a promising natural anti-inflammatory agent, particularly for conditions where multi-target modulation of COX, LOX, and inflammatory transcription factors is advantageous (Cao et al., 2010). Furthermore, prior reports indicating that Aster extracts attenuate NFκB/MAPK signaling pathways and reduce pro-inflammatory cytokines suggest that the COX-1 inhibition documented here is likely complemented *in vivo* by additional molecular mechanisms, potentially augmenting the overall anti-inflammatory efficacy of the plant.

Kaempferol-3,7-diglucopyranoside formed five hydrogen bonds with amino acids Asp197, Ala198, Asp300 (Elkomy et al., 2023), Lys200, and His299, along with H-π interactions with Ile235 (Khan et al., 2022); meanwhile, 1,4-dicaffeoylquinic acid created five hydrogen bonds with His201, Thr163 (Ahmed S. et al., 2022), Asp300, and His305, together with specific H–π contacts formed between the ligand aromatic rings and the Ile235 residue (Khan et al., 2022). Consistent with this, kaempferol-3,7-diglucopyranoside may be one of the components responsible for the extract’s anti-inflammatory activity. The work by Peng et al. indicated that dicaffeoylquinic acid could inhibit the release of major inflammatory mediators, including nitric oxide, prostaglandins, cytokines, and lipopolysaccharides (Wan et al., 2019). Molecular docking studies on both COX-1 and COX-2 enzymes supported these findings. Notably, most docked compounds bound to Arg120 (Kothayer et al., 2023), an important amino acid at the binding site. Dioctyl phthalate formed three hydrogen bonds with Tyr355 and Arg120, while kaempferol-3,7-diglucopyranoside formed two hydrogen bonds with Arg120 and Met522. Additionally, 1,4-Dicaffeoylquinic acid showed one hydrogen bond with Arg120, along with H-π interactions with Leu93. Docking results for COX-2 showed that stearidonic acid formed two hydrogen bonds with Arg513 and His90, while 5-caffeoylquinic acid formed five hydrogen bonds with Arg513 (Abdelkhalek et al., 2023). These results are predictive, and a future study could be performed to figure out the main mechanism for these compounds as COX-2 inhibitors. Overall, the molecular docking analysis reinforces the notion that the flavonoid and caffeoylquinic acid constituents of *S. squamatum* exhibit notable dual inhibitory activity against α-glucosidase and α-amylase on one hand and COX-1 on the other hand. Their bioactivity is primarily facilitated through a synergistic network of hydrogen bonding, hydrophobic forces, and π–π stacking interactions localized within the catalytic clefts of the target enzymes, thereby stabilizing enzyme–inhibitor complexes and impeding substrate access. These cumulative interactions underpin the extract’s observed antidiabetic activity and align well with computational and experimental evidence reported for other plant-derived inhibitors.​

## 5 Conclusion

*Symphyotrichum squamatum* is an annual weed belonging to the family Asteraceae. HPLC-PDA-ESI-MS/MS analysis identified 35 secondary metabolites, including the isolation of 3β-friedelinol, spinasterol, dioctyl phthalate, and kaempferol 3,7-diglucopyranoside. The hydro-alcoholic extract can be considered a potent antioxidant. The extract showed weak activity against human hepatocarcinoma cell line (HepG2) and human pancreatic cell line (Panc-1). *In vitro* assays revealed that the extract has potential as an anti-α-glucosidase and anti-α-amylase agent, making it a promising anti-diabetic candidate. It was also able to inhibit COX-1 enzyme, indicating anti-inflammatory activity. Furthermore, kaempferol-3,7-diglucopyranoside exhibited antidiabetic and anti-inflammatory effects by inhibiting α-glucosidase, α-amylase, and COX-1 enzymes. These findings align with molecular docking studies of the extract components against the tested enzymes.

## Data Availability

The original contributions presented in the study are publicly available. The data of C and H NMR can be found here https://doi.org/10.57992/nmrxiv.p167. The LC-MS data can be found here: doi:10.25345/C5ZK5612V.

## Glossary

ABTS: 2,2′-azino-bis(3-ethylbenzothiazoline-6-sulfonic acid)
ADMET: Absorption, Distribution, Metabolism, Excretion, and Toxicity
**AlCl**_**3**_: Aluminum chloride
ANOVA: One-way Analysis of Variance
AsA: Ascorbic acid
ATCC: American Type Culture Collection
COX-1: Cyclooxygenase-1
COX-2: Cyclooxygenase-2
DMSO: Dimethyl sulfoxide
DPPH: 2,2-diphenyl-1-picrylhydrazyl
EO: Essential oil
GA: Gallic acid
GAE: Gallic acid equivalents
HPLC: High-performance liquid chromatography
HPLC-PDA-ESI-MS/MS: High-performance liquid chromatography coupled with photodiode array detection and electrospray ionization tandem mass spectrometry
HSD: Honestly Significant Difference (Tukey’s test)
**IC**_**50**_: Half-maximal inhibitory concentration
LC/MS: Liquid chromatography–mass spectrometry
m/z: Mass-to-charge ratio
MM/GBSA ΔG: Molecular Mechanics/Generalized Born Surface Area binding free energy
MOE: Molecular Operating Environment
MTT: 3-(4,5-dimethylthiazol-2-yl)-2,5-diphenyltetrazolium bromide
PDB: Protein Data Bank
PE: Polyethylene
pNPG: p-nitrophenyl-α-D-glucopyranoside
PP: Polypropylene
QE: Quercetin equivalents
RMSD: Root-mean-square deviation
RMSF: Root-mean-square fluctuation
RSD: Relative standard deviation
**SC**_**50**_: Scavenging concentration (50% inhibition)
SD: Standard deviation
S/N: Signal-to-noise ratio
SSAE: *Symphyotrichum squamatum* aerial part extract
UV: Ultraviolet
Ala (Ala198): Alanine
Arg (Arg120, Arg513): Arginine
Asp (Asp197, Asp300): Aspartic acid
Glu (Glu524): Glutamic acid
His (His90, His299): Histidine
Ile (Ile235): Isoleucine
**Leu (Leu93, Leu352**): Leucine
Lys (Lys200): Lysine
Met (Met522): Methionine
Ser (Ser530): Serine
Tyr (Tyr355, Tyr385): Tyrosine
