# Supplementary material for: Phytochemical profiling and multi-target pharmacological evaluation of Symphyotrichum squamatum unveils its potential as a source of antidiabetic and anti-inflammatory agents
Source: Front Chem. 2026 Apr 13;14:1734411. doi: 10.3389/fchem.2026.1734411 (PMC13111398; doi:10.3389/fchem.2026.1734411)
Supplement: Supplementary file 1 [file DataSheet1.docx]

**Phytochemical Profiling and Multi-Target Pharmacological Evaluation of *Symphyotrichum squamatum* Unveils Its Potential as a Source of Antidiabetic and Anti-Inflammatory Agents** Mohammed Hassan^1^, Islam Mostafa^1,2,3,*^, Ahmed Saad^4,*^, Ahmed S. AbdelKhalek^5^, Mahmoud M. Elaasser^6^, Zepeng Yin^7,8^, Mahmoud Sitohy^4^, Mohamed El-Sadek ^5^, Omer I. Fantoukh^9^, Assem M. El-Shazly^1,10,^*, Mona Fekry ^1,^*

^1^ Department of Pharmacognosy, Faculty of Pharmacy, Zagazig University, Zagazig 44519, Egypt. ^2^ Pharmaceutical services center, Faculty of Pharmacy, Zagazig University, Zagazig 44519, Egypt.

^3^ Clinical Pharmacy Program, Medical Sector, Zagazig National University, Tenth of Ramadan City, Egypt.

^4^ Department of Biochemistry, Faculty of Agriculture, Zagazig University, Zagazig 44511, Egypt. [ahmedm4187@gmail.com](mailto:ahmedm4187@gmail.com)

^5^ Department of Medicinal Chemistry, Faculty of Pharmacy, Zagazig University, Zagazig 44519, Egypt.

^6^ Regional Center for Mycology and Biotechnology, Al-Azhar University, Cairo 11751, Egypt.

^7^ Key laboratory of fruit postharvest biology of Liaoning Province, Shenyang 110866, China.

^8^ College of Horticulture, Shenyang Agricultural University, Shenyang 110866, China.

^9^ Department of Pharmacognosy, College of Pharmacy, King Saud University, Riyadh 11451, Saudi Arabia.

^10^ Faculty of Pharmacy, El Saleheya El Gadida University, El Saleheya El Gadida 44813, Egypt.

**Supplemental data 1**: Spectroscopic data of the isolated compounds from petroleum ether and ethyl acetate fractions of *Symphyotrichum squamatum*

Compound 1: ^1^H-NMR (400 MHz, CDCl_3_) and ^13^C-NMR (100 MHz, CDCl_3_) data is represented in Table S1.

Compound 2: ^1^H-NMR (400 MHz, CDCl_3_) and ^13^C-NMR (100 MHz, CDCl_3_) data is represented in Table S1.

Compound 3: ^1^H-NMR (400 MHz, CDCl_3_) and ^13^C-NMR (100 MHz, CDCl_3_) data is represented in Table S2.

Compound 4: UV: λ_max_ (MeOH) nm is represented in Table S3. ^1^H-NMR (400 MHz, CD_3_OD) and ^13^C-NMR (100 MHz, CD_3_OD) data is represented in Table S4.

Table S1. The ^1^H-NMR and ^13^C-NMR spectral data of Compounds 1 and 2:

| Compound 2 | | Compound 1 | | | C-No. |
| --- | --- | --- | --- | --- | --- |
| δ C | δ H | δ C | δ H | |  |
| 37.2 | 1.31(m) | 19.84 | 1.34 (m) | 1 | |
| 31.6 | 1.31(m) | 37.23 | 1.34 (m) | 2 | |
| 71.1 | 3.59 (t) | 72.85 | 3.83 (t) | 3 | |
| 38.1 | 1.26 (m) | 53.12 | 1.17 (m) | 4 | |
| 40.4 | 1.26 (m) | 38.48 | --- | 5 | |
| 29.7 | 1.04 (m) | 41.69 | 1.17 (m) | 6 | |
| 117.5 | 5.16 (m) | 17.67 | 1.34 (m) | 7 | |
| 139.6 | --- | 52.77 | 1.28 (m) | 8 | |
| 49.6 | 1.25 (m) | 37.54 | --- | 9 | |
| 34.3 | --- | 61.37 | 1.04 (m) | 10 | |
| 21.6 | 1.04 (m) | 35.68 | 1.17 (m) | 11 | |
| 39.6 | 1.04 (m) | 30.76 | 1.28 (m) | 12 | |
| 43.4 | --- | 38.48 | --- | 13 | |
| 55.2 | 1.25 (m) | 39.50 | --- | 14 | |
| 23.1 | 1.04 (m) | 32.39 | 1.28 (m) | 15 | |
| 28.6 | 1.02 (m) | 36.20 | 1.28 (m) | 16 | |
| 56.0 | 1.25 (m) | 30.17 | --- | 17 | |
| 12.2 | 0.56 (s) | 42.94 | 1.5 (m) | 18 | |
| 13.1 | 0.87 (s) | 35.31 | 1.4 (m) | 19 | |
| 40.9 | 1.25 (m) | 28.29 | --- | 20 | |
| 21.4 | 1.04 (s) | 32.88 | 1.28 (m) | 21 | |
| 138.2 | 5.16 (m) | 39.26 | 1.4 (m) | 22 | |
| 129.5 | 5.03 (m) | 11.13 | 1.04 (s) | 23 | |
| 51.3 | 1.31 (m) | 14.76 | 0.78 (s) | 24 | |
| 32.0 | 1.25 (m) | 18.16 | 0.86 (s) | 25 | |
| 21.2 | 0.85 (s) | 20.23 | 0.98 (s) | 26 | |
| 19.1 | 0.80 (s) | 18.62 | 1.04 (s) | 27 | |
| 25.5 | 1.04 (m) | 32.20 | 1.17 (s) | 28 | |
| 12.4 | 0.80 (s) | 35.00 | 0.97 (s) | 29 | |
| --- | --- | 31.91 | 1.04 (s) | 30 | |

Table S2. The ^1^H-NMR and ^13^C-NMR spectral data of Compound 3

| C-No. | δ H | δ C |
| --- | --- | --- |
| C=O | --- | 167.9 |
| 1, 2 | --- | 132.6 |
| 3, 6 | 7.69 (d, J=4) | 131.1 |
| 4, 5 | 7.53(d, J=4) | 129.0 |
| 1′, 1′′ | 4.22 (dd) | 68.4 |
| 2′, 2′′ | 1.68 (m) | 38.9 |
| 3′-6′, 3′′-6′′ | 1.34- 1.44 (m) | 23.2 – 30.5 |
| 7′, 7′′ | 0.93 (t) | 14.2 |
| 8′, 8′′ | 0.91 (t) | 11.1 |

Table S3. The UV spectral data of Compound 4

| Shifting reagents | λmax (nm) | |
| --- | --- | --- |
|  | **Band I** | **Band II** |
| MeOH  MeOH + NaOMe  MeOH + AlCl_3_  MeOH + AlCl_3_ + HCl  MeOH+ NaOAc  MeOH+ NaOAc + H_3_BO_3_ | 377  410  428  427  377  377 | 264  268  268  267  264  264 |

Table S4. The ^1^H-NMR and ^13^C-NMR spectral data of Compound 4.

| δ C | δ H | C no. |
| --- | --- | --- |
| 159.1  135.4  179.9  163.1  99.9  165.9  94.8  158.5  105.8  122.8  132.3  116.1  161.6  104.0  75.7  78.4  71.3  78.0  62.6  101.8  74.8  78.6  71.1  78.3  62.4 | 6.17 (d, J=2.4)  6.39 (d, J=2)  8.06 (d, J=8.8)  6.89 (d, J=8.8)  5.47 (d, J=6.8)  3.33 (m)  3.33 (m)  3. 12 (m)  3. 12 (m)  3.53 m, 3.33 m  5.03 (d, J=6.8)  3.33 (m)  3.40 (m)  3.12 (m)  3.33 (m)  3.53 m, 3.46 m | 2  3  4  5  6  7  8  9  10  1'  2', 6'  3', 5'  4'  1''  2''  3''  4''  5''  6''  1'''  2'''  3'''  4'''  5'''  6''' |

| A  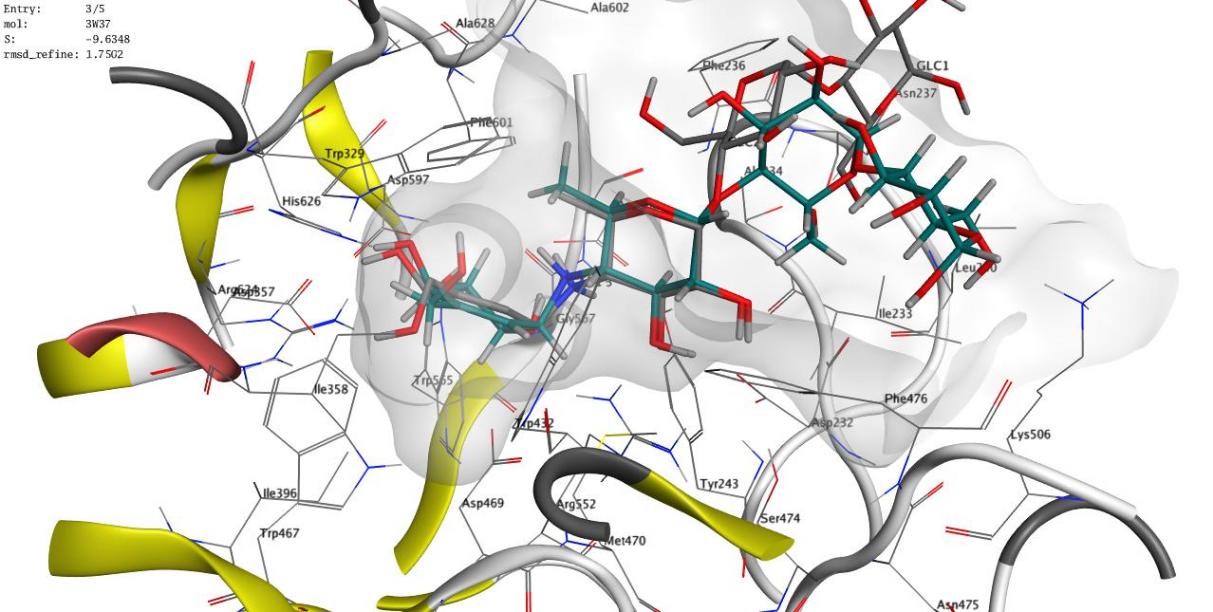 | **B**  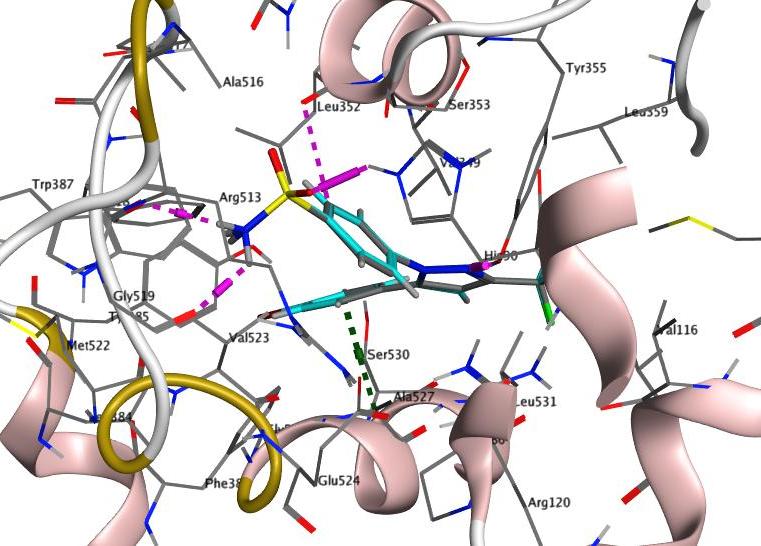 |
| --- | --- |

**Figure S1.** Validation of docking procedure at α-glucosidase (PDB ID: 3W37 **A**), and COX-2 (PDB ID: 1CX2) B binding sites.

**Figure S2.** SC_50_ of the hydroalcoholic extract of aerial parts extract of Symphyotrichum squamatum against DPPH free radicals.

**Figure S3.** SC_50_ of the hydroalcoholic extract of aerial parts extract of Symphyotrichum squamatum against ABTS free radicals.
